# Supplementary material for: Assessing population dynamics in the Central Salish Sea, Pacific Northwest Coast of North America
Source: PLoS One. 2023 Aug 3;18(8):e0285021. doi: 10.1371/journal.pone.0285021 (PMC10399822; doi:10.1371/journal.pone.0285021)
Supplement: S1 Appendix — (DOCX) [file pone.0285021.s001.docx]

Appendix A.

| Sample # | Site | 14C Age BP | ± Error | ΔR | Material | Reference |
| --- | --- | --- | --- | --- | --- | --- |
| Beta-123526 | 45SJ1 | 1220 | 40 |  | Charcoal | Stein et al. 2003 |
| Beta-123523 | 45SJ1 | 1240 | 50 |  | Charcoal | Stein et al. 2003 |
| Beta-123529 | 45SJ1 | 1500 | 40 |  | Charcoal | Stein et al. 2003 |
| Beta-123525 | 45SJ1 | 1540 | 100 |  | Charcoal | Stein et al. 2003 |
| Beta-123527 | 45SJ1 | 1610 | 40 |  | Charcoal | Stein et al. 2003 |
| USGS-20 | 45SJ1 | 2270 | 75 |  | Charcoal | Thompson 1978 |
| Beta-123532 | 45SJ1 | 2490 | 50 |  | Charcoal | Stein et al. 2003 |
| Beta-123533 | 45SJ1 | 2510 | 40 |  | Charcoal | Stein et al. 2003 |
| Beta-123531 | 45SJ1 | 2530 | 40 |  | Charcoal | Stein et al. 2003 |
| USGS-21 | 45SJ1 | 2630 | 75 |  | Charcoal | Thompson 1978 |
| USGS-22 | 45SJ1 | 2660 | 50 |  | Charcoal | Thompson 1978 |
| USGS-79 | 45SJ1 | 2700 | 90 |  | Charcoal | Thompson 1978 |
| USGS-26 | 45SJ1 | 1770 | 70 | 400 | Marine Shell | Robinson and Thompson 1980 |
| USGS-25 | 45SJ1 | 1280 | 100 | 400 | Marine Shell | Robinson and Thompson 1980 |
| USGS-10 | 45SJ1 | 1540 | 80 | 400 | Marine Shell | Robinson and Thompson 1981 |
| Beta-119301 | 45SJ105 | 360 | 50 |  | Charcoal | Stein et al. 2003 |
| Beta-119295 | 45SJ105 | 470 | 90 |  | Charcoal | Stein et al. 2003 |
| Beta-119303 | 45SJ105 | 670 | 40 |  | Charcoal | Stein et al. 2003 |
| Beta-119304 | 45SJ105 | 850 | 50 |  | Charcoal | Stein et al. 2003 |
| Beta-119302 | 45SJ105 | 850 | 70 |  | Charcoal | Stein et al. 2003 |
| Beta-119298 | 45SJ105 | 880 | 50 |  | Charcoal | Stein et al. 2003 |
| Beta-119299 | 45SJ105 | 930 | 60 |  | Charcoal | Stein et al. 2003 |
| Beta-119305 | 45SJ105 | 1010 | 60 |  | Charcoal | Stein et al. 2003 |
| Beta-119306 | 45SJ105 | 1010 | 70 |  | Charcoal | Stein et al. 2003 |
| UW-24 | 45SJ105 | 1514 | 40 |  | Charcoal | Kidd 1969 |
| Beta-119300 | 45SJ105 | 1810 | 50 |  | Charcoal | Stein et al. 2003 |
| Beta-119297 | 45SJ105 | 1860 | 50 |  | Charcoal | Stein et al. 2003 |
| Beta-119296 | 45SJ105 | 1950 | 100 |  | Charcoal | Stein et al. 2003 |
| Beta-24923 | 45SJ11 | 560 | 50 |  | Charcoal | Wessen 1988 |
| Beta-24924 | 45SJ11 | 2840 | 60 |  | Charcoal | Wessen 1988 |
| Beta-259802 | 45SJ120 | 520 | 40 | 400 | Marine Shell | Taylor et al. 2011 |
| Beta-223240 | 45SJ124 | 650 | 40 | 0 | Marine Shell | Taylor et al. 2011 |
| D-AMS 005562 | 45SJ13 | 125 | 24 |  | Charcoal | Nelson et al. 2014 |
| Beta-38449 | 45SJ133 | 2545 | 155 |  | Bone wedge | Kenady 1991 |
| Beta-218142 | 45SJ147 | 610 | 50 | 0 | Marine Shell | Taylor et al. 2011 |
| Beta-210408 | 45SJ147 | 680 | 40 | 0 | Marine Shell | Taylor et al. 2011 |
| Beta-218140 | 45SJ147 | 810 | 60 | 0 | Marine Shell | Taylor et al. 2011 |
| Beta-210407 | 45SJ147 | 870 | 40 | 0 | Marine Shell | Taylor et al. 2011 |
| Beta-218144 | 45SJ147 | 880 | 40 | 0 | Marine Shell | Taylor et al. 2011 |
| Beta-218141 | 45SJ147 | 890 | 40 | 0 | Marine Shell | Taylor et al. 2011 |
| Beta-218145 | 45SJ147 | 890 | 40 | 0 | Marine Shell | Taylor et al. 2011 |
| Beta-218139 | 45SJ147 | 930 | 40 | 0 | Marine Shell | Taylor et al. 2011 |
| Beta-218147 | 45SJ147 | 940 | 40 | 0 | Marine Shell | Taylor et al. 2011 |
| Beta-218146 | 45SJ147 | 970 | 40 | 0 | Marine Shell | Taylor et al. 2011 |
| Beta-218138 | 45SJ147 | 980 | 50 | 0 | Marine Shell | Taylor et al. 2011 |
| Beta-218143 | 45SJ147 | 1000 | 60 | 400 | Marine Shell | Taylor et al. 2011 |
| Beta-223241 | 45SJ150 | 670 | 30 | 0 | Marine Shell | Taylor et al. 2011 |
| Beta-223242 | 45SJ150 | 710 | 50 | 0 | Marine Shell | Taylor et al. 2011 |
| Beta-183121 | 45SJ153 | 1920 | 70 |  | Charcoal | Kenady et al. 2004 |
| Beta-183122 | 45SJ153 | 2420 | 70 |  | Charcoal | Kenady et al. 2004 |
| Beta-186502 | 45SJ153 | 130 | 60 |  | Charcoal | Kenady et al. 2004 |
| Beta-170642 | 45SJ165 | 1250 | 60 |  | Charcoal | Walker 2003 |
| Beta-170643 | 45SJ165 | 2640 | 60 |  | Charcoal | Walker 2003 |
| Beta-203874 | 45SJ165 | 3220 | 40 |  | Tooth | Bard 2007 |
| Beta-203875 | 45SJ165 | 3400 | 40 |  | Bone | Bard 2007 |
| Beta-203876 | 45SJ165 | 2480 | 60 |  | Charcoal | Bard 2007 |
| Beta-203877 | 45SJ165 | 2430 | 50 |  | Charcoal | Bard 2007 |
| Beta-203878 | 45SJ165 | 3400 | 40 |  | Bone | Bard 2007 |
| Beta-203879 | 45SJ165 | 2980 | 40 |  | Tooth | Bard 2007 |
| Beta-203880 | 45SJ165 | 2140 | 40 |  | Bone | Bard 2007 |
| Beta-203869 | 45SJ165 | 1050 | 60 |  | Charcoal | Bard 2007 |
| Beta-203870 | 45SJ165 | 2050 | 40 |  | Bone | Bard 2007 |
| Beta-203872 | 45SJ165 | 2350 | 40 |  | Charcoal | Bard 2007 |
| Beta-203871 | 45SJ165 | 2220 | 40 |  | Charcoal | Bard 2007 |
| Beta-203873 | 45SJ165 | 3350 | 40 |  | Charcoal | Bard 2007 |
| Beta-168999 | 45SJ169 | 2550 | 60 |  | Charcoal | Walker 2003 |
| Beta-170644 | 45SJ169 | 300 | 50 |  | Charcoal | Walker 2003 |
| Beta-170645 | 45SJ169 | 2490 | 60 |  | Charcoal | Walker 2003 |
| Beta-170646 | 45SJ169 | 1710 | 60 |  | Charcoal | Walker 2003 |
| Beta-170647 | 45SJ169 | 2680 | 120 |  | Charcoal | Walker 2003 |
| Beta-170648 | 45SJ169 | 2310 | 60 |  | Charcoal | Walker 2003 |
| Beta-170649 | 45SJ169 | 2450 | 40 |  | Charcoal | Walker 2003 |
| Beta-170650 | 45SJ169 | 2490 | 90 |  | Charcoal | Walker 2003 |
| Beta-170651 | 45SJ169 | 2400 | 50 |  | Charcoal | Walker 2003 |
| Beta-170652 | 45SJ169 | 110 | 60 |  | Charcoal | Walker 2003 |
| Beta-170653 | 45SJ169 | 2570 | 60 |  | Charcoal | Walker 2003 |
| Beta-170654 | 45SJ169 | 2370 | 60 |  | Charcoal | Walker 2003 |
| Beta-170655 | 45SJ169 | 2060 | 40 |  | Charcoal | Walker 2003 |
| N/A | 45SJ185 | 2545 | 155 |  | Charcoal | Wessen p.c. |
| Beta-89083 | 45SJ186 | 820 | 150 |  | Charcoal | Kenady 1996 |
| Beta-210403 | 45SJ2 | 2270 | 40 | 401 | Marine Shell | Taylor et al. 2011 |
| Beta-210404 | 45SJ2 | 2550 | 40 | 401 | Marine Shell | Taylor et al. 2011 |
| Beta-282440 | 45SJ200 | 1500 | 40 |  | Charcoal | Trost 2010 |
| Beta-278610 | 45SJ200 | 1010 | 40 | 400 | Marine Shell | Stein et al. 2010 |
| Beta-306019 | 45SJ200 | 50 | 30 |  | Charcoal | Nelson 2011 |
| Beta-223243 | 45SJ201 | 1040 | 40 | 400 | Marine Shell | Taylor et al. 2011 |
| Beta-223244 | 45SJ201 | 1180 | 60 | 400 | Marine Shell | Taylor et al. 2011 |
| Beta-234092 | 45SJ202 | 1180 | 40 | 400 | Marine Shell | Taylor et al. 2011 |
| Beta-259812 | 45SJ225 | 730 | 40 | 0 | Marine Shell | Taylor et al. 2011 |
| Beta-234094 | 45SJ23 | 1580 | 40 | 400 | Marine Shell | Taylor et al. 2011 |
| Beta-259813 | 45SJ239 | 1950 | 40 | 400 | Marine Shell | Taylor et al. 2011 |
| WSU-3514 310,304fB1#1 | 45SJ24 OpA | 160 | 60 |  | Charcoal | Stein et al. 2003 |
| OS-66817 | 45SJ24 OpA | 295 | 35 |  | Charcoal | Daniels 2009 [Unpublished] |
| WSU-3153 310,304f2#26 | 45SJ24 OpA | 355 | 50 |  | Charcoal | Stein et al. 2003 |
| WSU-3515 310,304fD1#5 | 45SJ24 OpA | 370 | 70 |  | Charcoal | Stein et al. 2003 |
| QL-4153 | 45SJ24 OpA | 430 | 40 |  | Charcoal | Stein et al. 2003 |
| WSU-3516 310,304fD3#6 | 45SJ24 OpA | 450 | 50 |  | Charcoal | Stein et al. 2003 |
| OS-66819 | 45SJ24 OpA | 500 | 30 |  | Charcoal | Daniels 2009 [Unpublished] |
| WSU-3517 310,300fB1#9 | 45SJ24 OpA | 535 | 80 |  | Charcoal | Stein et al. 2003 |
| 1983#3 | 45SJ24 OpA | 580 | 70 |  | Charcoal | Stein et al. 2003 |
| 1983#4 | 45SJ24 OpA | 630 | 55 |  | Charcoal | Stein et al. 2003 |
| WSU-3518 310,300fB1#1 | 45SJ24 OpA | 670 | 70 |  | Charcoal | Stein et al. 2003 |
| 294,270fF#18 | 45SJ24 OpA | 680 | 135 |  | Charcoal | Stein et al. 2003 |
| QL-4154 | 45SJ24 OpA | 810 | 80 |  | Charcoal | Stein et al. 2003 |
| WSU-1208 | 45SJ24 OpA | 820 | 240 |  | Charcoal | Thompson 1978 |
| QL-4156 | 45SJ24 OpA | 830 | 70 |  | Charcoal | Stein et al. 2003 |
| WSU-3152 310,300fC#7 | 45SJ24 OpA | 885 | 65 |  | Charcoal | Stein et al. 2003 |
| QL-4157 | 45SJ24 OpA | 900 | 40 |  | Charcoal | Stein et al. 2003 |
| QL-4155 | 45SJ24 OpA | 1000 | 40 |  | Charcoal | Stein et al. 2003 |
| WSU-1207 | 45SJ24 OpA | 1030 | 240 |  | Charcoal | Thompson 1978 |
| WSU-3151 310,300fN#25 | 45SJ24 OpA | 1070 | 80 |  | Charcoal | Stein et al. 2003 |
| OS-66818 | 45SJ24 OpA | 1140 | 30 |  | Charcoal | Daniels 2009 [Unpublished] |
| 310,302fD1#1 | 45SJ24 OpA | 1150 | 90 |  | Charcoal | Stein et al. 2003 |
| OS-66795 | 45SJ24 OpA | 1160 | 30 | 460 | P. staminea | Daniels 2009 [Unpublished] |
| OS-66816 | 45SJ24 OpA | 1180 | 25 |  | Charcoal | Daniels 2009 [Unpublished] |
| 310,302fD2#6 | 45SJ24 OpA | 1250 | 70 |  | Charcoal | Stein et al. 2003 |
| OS-66792 | 45SJ24 OpA | 1330 | 25 | 380 | P. staminea | Daniels 2009 [Unpublished] |
| OS-66793 | 45SJ24 OpA | 1570 | 30 | 620 | P. staminea | Daniels 2009 [Unpublished] |
| WSU-3519 310,300fR3#4 | 45SJ24 OpA | 1585 | 70 |  | Charcoal | Stein et al. 2003 |
| 310,302fE1#1 | 45SJ24 OpA | 1690 | 60 |  | Charcoal | Stein et al. 2003 |
| OS-66796 | 45SJ24 OpA | 1820 | 25 | 420 | P. staminea | Daniels 2009 [Unpublished] |
| OS-66797 | 45SJ24 OpA | 1840 | 25 | 475 | P. staminea | Daniels 2009 [Unpublished] |
| OS-66794 | 45SJ24 OpA | 1980 | 30 | 455 | P. staminea | Daniels 2009 [Unpublished] |
| OS-67018 | 45SJ24 OpD | 740 | 25 |  | Charcoal | Daniels 2009 [Unpublished], Stein et al. 2011 |
| TGH008 | 45SJ24 OpD | 1170 | 70 |  | Charcoal | Stein et al. 2003 |
| 123I1 | 45SJ24 OpD | 1180 | 70 |  | Charcoal | Stein et al. 2003 |
| 130C1 | 45SJ24 OpD | 1230 | 60 |  | Charcoal | Stein et al. 2003 |
| TEF0077 | 45SJ24 OpD | 1300 | 80 |  | Charcoal | Stein et al. 2003 |
| 130H1 | 45SJ24 OpD | 1300 | 60 |  | Charcoal | Stein et al. 2003 |
| 130K1 | 45SJ24 OpD | 1300 | 50 |  | Charcoal | Stein et al. 2003 |
| OS-66976 | 45SJ24 OpD | 1345 | 75 |  | Charcoal | Stein et al. 2011 |
| 123A5 | 45SJ24 OpD | 1370 | 80 |  | Charcoal | Stein et al. 2003 |
| Beta-84219 (105T1C) | 45SJ24 OpD | 1400 | 90 |  | Mixed confier bole | Stein et al. 2003, Deo et al. 2004 |
| TGH009 | 45SJ24 OpD | 1410 | 80 |  | Charcoal | Stein et al. 2003 |
| TABD2 | 45SJ24 OpD | 1430 | 70 |  | Charcoal | Stein et al. 2003 |
| TEF0073 | 45SJ24 OpD | 1430 | 50 |  | Charcoal | Stein et al. 2003 |
| TAB004 | 45SJ24 OpD | 1460 | 50 |  | Charcoal | Stein et al. 2003 |
| 111W1 | 45SJ24 OpD | 1460 | 70 |  | Charcoal | Stein et al. 2003 |
| TCD002 | 45SJ24 OpD | 1470 | 70 |  | Charcoal | Stein et al. 2003 |
| Beta-84216 (105B7) | 45SJ24 OpD | 1470 | 90 |  | Conifer (not Pseudotsuga menziesii or Thuja plicata) | Stein et al. 2003, Deo et al. 2004 |
| TABC1 | 45SJ24 OpD | 1480 | 80 |  | Charcoal | Stein et al. 2003 |
| OS-66815 | 45SJ24 OpD | 1500 | 70 |  | Charcoal | Stein et al. 2011 |
| TAB003 | 45SJ24 OpD | 1600 | 60 |  | Charcoal | Stein et al. 2003 |
| 105O1 | 45SJ24 OpD | 1710 | 70 |  | Charcoal | Stein et al. 2003 |
| TAB007 | 45SJ24 OpD | 1740 | 60 |  | Charcoal | Stein et al. 2003 |
| 111P1 | 45SJ24 OpD | 1760 | 80 |  | Charcoal | Stein et al. 2003 |
| TGHB3 | 45SJ24 OpD | 1800 | 70 |  | Charcoal | Stein et al. 2003 |
| 111EE2 | 45SJ24 OpD | 1940 | 110 |  | Charcoal | Stein et al. 2003 |
| OS-66791 | 45SJ24 OpD | 2170 | 30 | 1020 | P. staminea | Daniels 2009 [Unpublished] |
| Beta-84220 | 45SJ24 OpD | 2210 | 70 | 401 | Unid. Marine Shell | Deo et al. 2004 |
| Beta-84218 | 45SJ24 OpD | 2370 | 70 | 401 | Unid. Marine Shell | Deo et al. 2004 |
| Gak-4944 | 45SJ25 | 1580 | 60 |  | Charcoal | Carlson 1976 |
| Gak-4933 | 45SJ25 | 2100 | 100 |  | Charcoal | Carlson 1976 |
| Beta-216372 | 45SJ251 | 710 | 40 | 0 | Marine Shell | Taylor et al. 2011 |
| Beta-210409 | 45SJ251 | 790 | 40 | 0 | Marine Shell | Taylor et al. 2011 |
| Beta-210410 | 45SJ251 | 790 | 40 | 0 | Marine Shell | Taylor et al. 2011 |
| Beta-216328 | 45SJ251 | 1140 | 40 | 400 | Marine Shell | Taylor et al. 2011 |
| Beta-119307 | 45SJ254 | 510 | 50 |  | Mixed confier bole | Stein et al. 2003, Deo et al. 2004 |
| Beta-119309 | 45SJ254 | 1310 | 40 |  | Charcoal | Stein et al. 2003 |
| Beta-119308 | 45SJ254 | 1410 | 40 |  | Charcoal | Stein et al. 2003 |
| CAMS-56446 | 45SJ254 | 2070 | 50 | 401 | Veneridae | Deo et al. 2004 |
| Beta-234095 | 45SJ26 | 580 | 40 | 400 | Marine Shell | Taylor et al. 2011 |
| Beta-223238 | 45SJ27 | 930 | 40 | 0 | Marine Shell | Taylor et al. 2011 |
| Beta-223239 | 45SJ27 | 1140 | 40 | 400 | Marine Shell | Taylor et al. 2011 |
| Tx-4018 | 45SJ274 | 278 | 60 |  | Charcoal | Benson 1981 |
| Tx-4017 | 45SJ274 | 2860 | 121 |  | Charcoal | Benson 1981 |
| Beta-223246 | 45SJ274 | 1140 | 70 | 400 | Marine Shell | Taylor et al. 2011 |
| Beta-223247 | 45SJ274 | 1330 | 40 | 400 | Marine Shell | Taylor et al. 2011 |
| Beta-223245 | 45SJ274 | 1470 | 40 | 400 | Marine Shell | Taylor et al. 2011 |
| Beta-223248 | 45SJ274 | 1670 | 70 | 400 | Marine Shell | Taylor et al. 2011 |
| Beta-223249 | 45SJ277 | 930 | 40 | 0 | Marine Shell | Taylor et al. 2011 |
| Beta-119315 | 45SJ278 | 690 | 90 |  | Thuja/Tsuga branch | Stein et al. 2003, Deo et al. 2004 |
| CAMS-56450 | 45SJ278 | 920 | 50 | 0 | Lottidae | Deo et al. 2004 |
| Beta-119313 | 45SJ278 | 1090 | 70 |  | P. menziesii branch | Stein et al. 2003, Deo et al. 2004 |
| Beta-119310 | 45SJ278 | 1210 | 50 |  | Conifer, mixed branch, bole | Stein et al. 2003, Deo et al. 2004 |
| Beta-119314 | 45SJ278 | 1210 | 60 |  | P. menziesii bole and bark | Stein et al. 2003, Deo et al. 2004 |
| Beta-123534 | 45SJ278 | 1270 | 50 |  | Charcoal | Stein et al. 2003 |
| CAMS-56448 | 45SJ278 | 1510 | 50 | 401 | Pelecypoda | Deo et al. 2004 |
| Beta-119311 | 45SJ278 | 1580 | 50 |  | Charcoal | Stein et al. 2003 |
| CAMS-56447 | 45SJ278 | 1610 | 50 | 401 | Mollusca | Deo et al. 2004 |
| Beta-123535 | 45SJ278 | 1770 | 40 |  | Charcoal | Stein et al. 2003 |
| Beta-119312 | 45SJ278 | 3190 | 110 |  | Charcoal | Stein et al. 2003 |
| CAMS-56449 | 45SJ278 | 3900 | 40 | 2300 | Saxidomus sp. | Deo et al. 2004 |
| Beta-223258 | 45SJ279 | 700 | 40 | 0 | Marine Shell | Taylor et al. 2011 |
| Beta-223251 | 45SJ279 | 960 | 40 | 0 | Marine Shell | Taylor et al. 2011 |
| Beta-223250 | 45SJ279 | 1090 | 40 | 400 | Marine Shell | Taylor et al. 2011 |
| Beta-223253 | 45SJ279 | 1340 | 40 | 400 | Marine Shell | Taylor et al. 2011 |
| Beta-223252 | 45SJ279 | 1700 | 40 | 400 | Marine Shell | Taylor et al. 2011 |
| Beta-119320 | 45SJ280 | 120 | 50 |  | Charcoal | Stein et al. 2005 |
| OS-66822 | 45SJ280 | 500 | 30 |  | Charcoal | Daniels 2009 [Unpublished] |
| OS-66823 | 45SJ280 | 665 | 30 |  | Charcoal | Daniels 2009 [Unpublished] |
| OS-68461 | 45SJ280 | 1160 | 25 | 160 | P. staminea | Daniels 2009 [Unpublished] |
| Beta-119317 | 45SJ280 | 1350 | 100 |  | Charcoal | Stein et al. 2005 |
| Beta-119318 | 45SJ280 | 1560 | 50 |  | P. menziesii branch | Deo et al. 2004 |
| Beta-119319 | 45SJ280 | 1580 | 50 |  | Conifer branch | Deo et al. 2004 |
| OS-68460 | 45SJ280 | 1840 | 25 | 890 | P. staminea | Daniels 2009 [Unpublished] |
| OS-84405 | 45SJ280 | 1930 | 25 | 401 | *P. staminea* | Taylor 2012 [Unpublished] |
| OS-84404 | 45SJ280 | 2090 | 30 | 401 | Bivalve | Taylor 2012 [Unpublished] |
| Beta-119322 | 45SJ280 | 2090 | 40 |  | Charcoal | Stein et al. 2005 |
| Beta-119323 | 45SJ280 | 2110 | 50 |  | Charcoal | Stein et al. 2005 |
| OS-45739 | 45SJ280 | 2130 | 30 | 401 | *Mytilus californianus* | Bovy 2005 |
| OS-89922 | 45SJ280 | 2140 | 35 | 401 | *Nucella* spp. | Taylor 2012 [Unpublished] |
| OS-89919 | 45SJ280 | 2150 | 30 | 401 | Polyplacophora | Taylor 2012 [Unpublished] |
| OS-42367 | 45SJ280 | 2160 | 30 | 401 | *Acme mitra* | Bovy 2005 |
| CAMS-56455 | 45SJ280 | 2170 | 50 | 401 | *Balanus* sp. | Deo et al. 2004 |
| OS-89923 | 45SJ280 | 2200 | 30 | 401 | *P. staminea* | Taylor 2012 [Unpublished] |
| Beta-119321 | 45SJ280 | 2200 | 50 |  | Pseudotsuga menziesii | Deo et al. 2004 |
| CAMS-56453 | 45SJ280 | 2240 | 50 | 401 | *Stronglyocentrotus* sp. | Deo et al. 2004 |
| OS-89920 | 45SJ280 | 2280 | 25 | 401 | *Balanus spp.* | Taylor 2012 [Unpublished] |
| OS-42736 | 45SJ280 | 2280 | 25 | 401 | Bivalve | Bovy 2005 |
| OS-88418 | 45SJ280 | 2290 | 25 | 401 | Bivalve | Taylor 2012 [Unpublished] |
| OS-42735 | 45SJ280 | 2300 | 30 | 401 | Bivalve | Bovy 2005 |
| OS-42362 | 45SJ280 | 2310 | 30 | 401 | *Tresus* sp. | Bovy 2005 |
| OS-42361 | 45SJ280 | 2320 | 30 | 401 | Polyplacophora | Bovy 2005 |
| OS-42278 | 45SJ280 | 2330 | 30 | 401 | Mollusca | Bovy 2005 |
| OS-42364 | 45SJ280 | 2330 | 30 | 401 | Polyplacophora | Bovy 2005 |
| CAMS-56454 | 45SJ280 | 2330 | 50 | 401 | Bivalve | Deo et al. 2004 |
| OS-89921 | 45SJ280 | 2340 | 30 | 401 | Polyplacophora | Taylor 2012 [Unpublished] |
| OS-42360 | 45SJ280 | 2340 | 30 | 401 | *P. staminea* | Bovy 2005 |
| OS-45738 | 45SJ280 | 2340 | 30 | 401 | *P. staminea* | Bovy 2005 |
| OS-45737 | 45SJ280 | 2360 | 30 | 401 | *P. staminea* | Bovy 2005 |
| OS-42363 | 45SJ280 | 2360 | 30 | 401 | Polyplacophora | Bovy 2005 |
| Beta-119316 | 45SJ280 | 2360 | 50 |  | *Thuja/Tsuga* branch | Deo et al. 2004 |
| OS-42365 | 45SJ280 | 2380 | 35 | 401 | *P. staminea* | Bovy 2005 |
| OS-42366 | 45SJ280 | 2400 | 30 |  | Bivalve | Bovy 2005 |
| OS-42279 | 45SJ280 | 2450 | 35 | 401 | *P. staminea* | Bovy 2005 |
| Beta-119324 | 45SJ280 | 2640 | 40 |  | Conifer branch | Deo et al. 2004 |
| CAMS-56451 | 45SJ280 | 3150 | 40 | 401 | *P. staminea* | Deo et al. 2004 |
| CAMS-56452 | 45SJ280 | 3320 | 50 | 600 | Gastropoda | Deo et al. 2004 |
| Beta-193785 | 45SJ280 | 3430 | 40 |  | *Uria* cf. *aalge* humerus | Stein et al. 2005 |
| Beta-89084 | 45SJ281 | 830 | 80 | 0 | Marine Shell | Kenady 1996 |
| Beta-223254 | 45SJ282 | 770 | 40 | 0 | Marine Shell | Taylor et al. 2011 |
| Beta-223255 | 45SJ282 | 2020 | 50 | 400 | Marine Shell | Taylor et al. 2011 |
| Beta-223236 | 45SJ3 | 820 | 40 | 0 | Marine Shell | Taylor et al. 2011 |
| Beta-223237 | 45SJ3 | 820 | 40 | 0 | Marine Shell | Taylor et al. 2011 |
| Beta-223235 | 45SJ3 | 2250 | 40 | 400 | Marine Shell | Taylor et al. 2011 |
| Beta-223234 | 45SJ3 | 2280 | 40 | 400 | Marine Shell | Taylor et al. 2011 |
| Beta-259808 | 45SJ307 | 1120 | 40 | 400 | Marine Shell | Taylor et al. 2011 |
| Beta-234926 | 45SJ324 | 1110 | 40 | 400 | Marine Shell | Baldwin 2007 |
| Beta-234927 | 45SJ324 | 1090 | 40 | 400 | Marine Shell | Baldwin 2007 |
| Beta-235237 | 45SJ324 | 1190 | 40 | 400 | Marine Shell | Baldwin 2007 |
| Beta-267090 | 45SJ364 | 940 | 40 | 0 | Marine Shell | Taylor et al. 2011 |
| Beta-30941 | 45SJ369 | 1560 | 70 |  | Charcoal | Kenady 1991 |
| Beta-216329 | 45SJ407 | 2570 | 40 | 400 | Marine Shell | Taylor et al. 2011 |
| Beta-210405 | 45SJ407 | 2650 | 40 | 400 | Marine Shell | Taylor et al. 2011 |
| Beta-210406 | 45SJ407 | 2680 | 40 | 400 | Marine Shell | Taylor et al. 2011 |
| Beta-84877 | 45SJ414 | 4750 | 60 |  | Charcoal | Kenady et al. 2002 |
| Beta-84878 | 45SJ414 | 3750 | 60 |  | Charcoal | Kenady et al. 2002 |
| Beta-384722 | 45SJ438 | 940 | 30 | 390 | Urchin | Arthur and Mather 2014 |
| Beta-218150 | 45SJ450 | 670 | 40 | 0 | Marine Shell | Taylor et al. 2011 |
| Beta-218153 | 45SJ450 | 830 | 60 | 0 | Marine Shell | Taylor et al. 2011 |
| Beta-218152 | 45SJ450 | 840 | 60 | 0 | Marine Shell | Taylor et al. 2011 |
| Beta-218151 | 45SJ450 | 900 | 40 | 0 | Marine Shell | Taylor et al. 2011 |
| Beta-210411 | 45SJ450 | 1110 | 40 | 400 | Marine Shell | Taylor et al. 2011 |
| Beta-218154 | 45SJ450 | 1600 | 40 | 400 | Marine Shell | Taylor et al. 2011 |
| Beta-218149 | 45SJ450 | 1760 | 40 | 400 | Marine Shell | Taylor et al. 2011 |
| Beta-218148 | 45SJ450 | 1890 | 60 | 400 | Marine Shell | Taylor et al. 2011 |
| Beta-210412 | 45SJ450 | 2130 | 40 | 400 | Marine Shell | Taylor et al. 2011 |
| Beta-210413 | 45SJ451 | 610 | 40 | 0 | Marine Shell | Taylor et al. 2011 |
| Beta-218156 | 45SJ451 | 750 | 40 | 0 | Marine Shell | Taylor et al. 2011 |
| Beta-218155 | 45SJ451 | 750 | 40 | 0 | Marine Shell | Taylor et al. 2011 |
| Beta-210414 | 45SJ451 | 820 | 40 | 0 | Marine Shell | Taylor et al. 2011 |
| Beta-218256 | 45SJ453 | 760 | 40 | 0 | Marine Shell | Taylor et al. 2011 |
| Beta-223257 | 45SJ460 | 910 | 40 | 0 | Marine Shell | Taylor et al. 2011 |
| Beta-223259 | 45SJ461 | 700 | 40 | 0 | Marine Shell | Taylor et al. 2011 |
| Beta-259803 | 45SJ47 | 700 | 40 | 0 | Marine Shell | Taylor et al. 2011 |
| Beta-259806 | 45SJ481 | 600 | 40 | 0 | Marine Shell | Taylor et al. 2011 |
| Beta-259809 | 45SJ483 | 720 | 40 | 0 | Marine Shell | Taylor et al. 2011 |
| Beta-259805 | 45SJ507 | 470 | 40 | 400 | Marine Shell | Taylor et al. 2011 |
| Beta-267088 | 45SJ509 | 960 | 40 | 0 | Marine Shell | Taylor et al. 2011 |
| Beta-333218 | 45SJ540 | 3960 | 30 | 0 | Marine Shell | Wessen 2013 |
| Beta-333219 | 45SJ540 | 1400 | 30 | 400 | Marine Shell | Wessen 2013 |
| Beta-234093 | 45SJ6 | 810 | 40 | 0 | Marine Shell | Taylor et al. 2011 |
| Beta-267091 | 45SJ60 | 720 | 40 | 0 | Marine Shell | Taylor et al. 2011 |
| Beta-259815 | 45SJ61 | 730 | 40 | 0 | Marine Shell | Taylor et al. 2011 |
| Beta-259816 | 45SJ70 | 1030 | 40 | 400 | Marine Shell | Taylor et al. 2011 |
| Beta-267092 | 45SJ70 | 2160 | 60 | 400 | Marine Shell | Taylor et al. 2011 |
| Beta-259814 | 45SJ71 | 980 | 40 | 0 | Marine Shell | Taylor et al. 2011 |
| Beta-259807 | 45SJ72 | 1030 | 40 | 400 | Marine Shell | Taylor et al. 2011 |
| Gx-5653 | 45SJ84 | 2570 | 140 |  | Bone | Bailey 1981 |
| Gx-5652 | 45SJ84 | 2785 | 125 |  | Bone | Bailey 1981 |
| Beta-259810 | 45SJ89 | 770 | 40 | 0 | Marine Shell | Taylor et al. 2011 |
| Beta-259811 | 45SJ9 | 910 | 40 | 0 | Marine Shell | Taylor et al. 2011 |
| Beta-267089 | 45SJ95 | 620 | 40 | 0 | Marine Shell | Taylor et al. 2011 |
| Beta-249323 | 45SK139 | 860 | 40 |  | Charcoal | Walker et. al. 2009 |
| Beta-249324 | 45SK139 | 1020 | 40 |  | Charcoal | Walker et. al. 2009 |
| Beta-249320 | 45SK139 | 450 | 40 |  | Charcoal | Walker et. al. 2009 |
| Beta-249322 | 45SK139 | 3090 | 40 |  | Charcoal | Walker et. al. 2009 |
| Beta-249321 | 45SK139 | 240 | 40 |  | Charcoal | Walker et. al. 2009 |
| Beta-209900 | 45SK144 | 1380 | 90 |  | Charcoal | Campbell 2013 (Database) |
| Beta-(A) | 45SK155 | 2040 | 100 |  | Charcoal | Wessen and Waterhouse 1987 |
| WSU-4242 | 45SK156 | 1000 | 129 |  | Basket | Rice 1983 |
| Beta-(B) | 45SK158 | 1000 | 70 |  | Charcoal | Wessen and Waterhouse 1987 |
| WSU-3137 | 45SK171 | 475 | 65 |  | Charcoal | Mierendorf 1986 |
| Beta-31524 | 45SK200 | 6000 | 500 |  | Charcoal | Larson p.c. |
| GaK-4933 | 45SK25 | 2180 | 100 |  | Charcoal | Thompson 1978 |
| Beta-239564 | 45SK258 | 3220 | 40 |  | Peat | Kopperl 2011 |
| Beta-239565 | 45SK258 | 2050 | 40 |  | Charcoal | Kopperl 2011 |
| Beta-239566 | 45SK258 | 2190 | 40 |  | Charcoal | Kopperl 2011 |
| Beta-239567 | 45SK258 | 2070 | 40 |  | Charcoal | Kopperl 2011 |
| UW-191 | 45SK33 | 266 | 50 |  | Charcoal | Blukis-Onat 1980 |
| USGS-17 | 45SK33 | 900 | 90 |  | Marine Shell | Blukis-Onat 1980 |
| UW-194 | 45SK33 | 738 | 50 |  | Marine Shell | Blukis-Onat 1980 |
| UW-190 | 45SK33 | 970 | 69 |  | Charcoal | Blukis-Onat 1980 |
| UW-195 | 45SK33 | 1126 | 110 | 400 | Marine Shell | Blukis-Onat 1980 |
| UW-196 | 45SK33 | 1230 | 150 | 400 | Marine Shell | Blukis-Onat 1980 |
| UW-148 | 45SK33 | 426 | 75 |  | Charcoal | Blukis-Onat 1980 |
| UW-149 | 45SK33 | 1024 | 90 |  | Charcoal | Blukis-Onat 1980 |
| UW-150 | 45SK33 | 1045 | 100 |  | Charcoal | Blukis-Onat 1980 |
| UW-151 | 45SK33 | 1116 | 85 |  | Charcoal | Blukis-Onat 1980 |
| GX-3627 | 45SK37 | 400 | 120 |  | Charcoal | Thompson 1978 |
| GX-3629 | 45SK37 | 430 | 115 |  | Charcoal | Thompson 1978 |
| GX-3630 | 45SK37 | 520 | 85 |  | Charcoal | Thompson 1978 |
| GX-3628 | 45SK37 | 640 | 115 |  | Charcoal | Thompson 1978 |
| USGS-11 | 45SK37 | 1140 | 70 | 400 | Marine Shell | Robinson and Thompson 1981 |
| USGS-10 | 45SK37 | 1540 | 80 | 400 | Marine Shell | Robinson and Thompson 1981 |
| QL-12 | 45SK37 | 1230 | 50 | 400 | Marine Shell | Robinson and Thompson 1981 |
| QL-15 | 45SK41 | 2180 | 70 |  | Charcoal | Thompson 1978 |
| QL-14 | 45SK41 | 2680 | 50 |  | Charcoal | Thompson 1978 |
| Beta-259804 | 45SK421 | 1490 | 40 | 400 | Marine Shell | Taylor et al. 2011 |
| Beta-280215 | 45SK43 | 1160 | 50 |  | Charcoal | Nelson et al. 2010 |
| Beta-280216 | 45SK43 | 2690 | 60 |  | Charcoal | Nelson et al. 2010 |
| Beta-280217 | 45SK43 | 1400 | 40 |  | Charcoal | Nelson et al. 2010 |
| Beta-204038 | 45SK43 | 690 | 70 | 390 | Marine Shell | Campbell 2013 (Database) |
| Beta-286275 | 45SK438 | 290 | 40 |  | Charcoal | Bush et. al. 2011 |
| Beta-243029 | 45SK46 | 1160 | 40 | 401 | Marine Shell | Mather 2009 [Unpublished] |
| Beta-243028 | 45SK46 | 2910 | 50 | 401 | Marine Shell | Mather 2009 [Unpublished] |
| Beta-204039 | 45SK46 | 3060 | 60 | 401 | Saxidomus | Mather 2009 [Unpublished] |
| Beta-243030 | 45SK46 | 3240 | 70 | 600 | Marine Shell | Mather 2009 [Unpublished] |
| N-1822 | 45SK51 | 1160 | 80 |  | Charcoal | Thompson 1978 |
| N-1823 | 45SK51 | 1190 | 75 |  | Charcoal | Thompson 1978 |
| USGS-12 | 45SK53 | 1530 | 90 | 400 | Marine Shell | Robinson and Thompson 1981 |
| USGS-37 | 45SK53 | 675 | 55 |  | Charcoal | Robinson and Thompson 1981 |
| USGS-8 | 45SK57 | 1440 | 70 | 400 | Marine Shell | Robinson and Thompson 1981 |
| USGS-9 | 45SK57 | 3400 | 80 |  | Marine Shell | Robinson and Thompson 1981 |
| USGS-15 | 45SK57 | 1380 | 100 | 400 | Marine Shell | Robinson and Thompson 1981 |
| UW-170 | 45SK59 | 670 | 75 |  | Cedar basket | Munsell 1976 |
| USGS-18 | 45SK59 | 800 | 70 |  | Charcoal | Thompson 1978 |
| USGS-58 | 45SK59 | 980 | 45 |  | Charcoal | Thompson 1978 |
| USGS-57 | 45SK59 | 1270 | 60 |  | Charcoal | Thompson 1978 |
| QL-13 | 45SK7 | 600 | 100 |  | Charcoal | Thompson 1978 |
| USGS-13 | 45SK77 | 1340 | 110 |  | Charcoal | Thompson 1978 |
| USGS-13B | 45SK77 | 1080 | 50 |  | Charcoal | Thompson 1978 |
| QL-18 | 45SK81 | 1950 | 60 | 400 | Marine Shell | Robinson and Thompson 1981 |
| USGS-427 | 45SK99 | 1560 | 45 | 400 | Marine Shell | Blukis-Onat 1980 |
| USGS-428 | 45SK99 | 1630 | 50 | 400 | Marine Shell | Blukis-Onat 1980 |
| WSU-1472 | 45SK99 | 1220 | 70 |  | Charcoal | Blukis-Onat 1980 |
| Beta-294108 | 45WH1 | 40 | 30 |  | Mammal Bone | Dubeau 2012 [Unpublished] |
| Beta-298339 | 45WH1 | 890 | 40 | 420 | Marine Shell | Campbell 2011 |
| UGAMS-03342 | 45WH1 | 1470 | 25 | 401 | Thais lamellosa | Rorabaugh 2009 [Unpublished] |
| D-AMS-003682 | 45WH1 | 1127 | 20 | 0 | Thais lamellosa | Rorabaugh 2017 |
| D-AMS-003683 | 45WH1 | 1136 | 22 | 0 | Thais lamellosa | Rorabaugh 2017 |
| UW, Cat. #1561 | 45WH1 | 960 | 200 |  | Charcoal | Blodgett 1975 |
| Beta-294109 | 45WH1 | 1120 | 30 |  | Mammal Bone | Dubeau 2012 |
| Beta-292829 | 45WH1 | 1230 | 40 |  | Holodiscus discolor | Palmer 2015 [Unpublished] |
| UW, Cat. #1149 | 45WH1 | 1300 | 200 |  | Charcoal | Blodgett 1975 |
| D-AMS-003681 | 45WH1 | 2050 | 25 | 401 | Thais lamellosa | Rorabaugh 2013 |
| UW, Cat. #1250 | 45WH1 | 1640 | 200 |  | Charcoal | Blodgett 1975 |
| Beta-299323 | 45WH1 | 2860 | 50 | 454 | Marine Shell | Palmer 2015 [Unpublished] |
| Beta-307547 | 45WH1 | 2970 | 30 | 420 | Marine Shell | Palmer 2015 [Unpublished] |
| WWSC, Cat. #633 | 45WH1 | 2340 | 200 |  | Charcoal | Blodgett 1975; Grabert and Larsen 1975 |
| D-AMS-003684 | 45WH1 | 3461 | 25 | 800 | Thais lamellosa | Rorabaugh 2017 |
| Beta-279605 | 45WH1 | 3140 | 50 | 454 | Thais lamellosa | Taber 2010 [Unpublished] |
| Beta-292828 | 45WH1 | 2420 | 30 |  | Cervus elaphus | Palmer 2015 [Unpublished] |
| Beta-299324 | 45WH1 | 2810 | 30 | 454 | Marine Shell | Palmer 2015 [Unpublished] |
| Beta-279606 | 45WH1 | 3270 | 50 | 454 | Thais lamellosa | Taber 2010 [Unpublished] |
| UGAMS-04047 | 45WH1 | 3340 | 30 | 454 | Thais lamellosa | Rorabaugh 2009 |
| RL-272 | 45WH1 | 2630 | 240 |  | Charcoal | Blodgett 1975 |
| Beta-4109 | 45WH100 | 2410 | 60 |  | Charcoal | Grabert and Griffin 1983 |
| UW-344 | 45WH11 | 1945 | 98 |  | Charcoal | Gaston and Grabert 1975 |
| Beta-4107 | 45WH110 | 420 | 50 |  | Charcoal | Grabert and Griffin 1983 |
| Beta-4106 | 45WH111 | 1590 | 60 |  | Charcoal | Grabert and Griffin 1983 |
| Beta-4105 | 45WH111 | 460 | 50 |  | Charcoal | Grabert and Griffin 1983 |
| Beta-4108 | 45WH114 | 2040 | 190 |  | Charcoal | Grabert and Griffin 1983 |
| GX-32130 | 45WH17 | 930 | 70 | 401 | Sea lion | Tierney 2012:73 [Unpublished] |
| UW-461 | 45WH17 | 350 | 50 |  | Charcoal | Montgomery 1979 |
| D-AMS-003686 | 45WH17 | 1293 | 22 | 401 | Thais lamellosa | Rorabaugh 2017 |
| UW-462 | 45WH17 | 580 | 60 |  | Charcoal | Montgomery 1979 |
| D-AMS-003685 | 45WH17 | 1120 | 28 | 0 | Thais lamellosa | Rorabaugh 2017 |
| UW-458 | 45WH17 | 830 | 60 |  | Charcoal | Montgomery 1979 |
| UW-457 | 45WH17 | 2370 | 70 |  | Charcoal | Montgomery 1979 |
| D-AMS-003687 | 45WH17 | 3185 | 24 | 401 | Thais lamellosa | Rorabaugh 2017 |
| UW-332 | 45WH17 | 2875 | 65 |  | Charcoal | Gaston 1975 |
| UW-463 | 45WH17 | 2715 | 55 |  | Charcoal | Montgomery 1979 |
| UW-459 | 45WH17 | 2830 | 65 |  | Charcoal | Montgomery 1979 |
| UW-460 | 45WH17 | 3015 | 65 |  | Charcoal | Montgomery 1979 |
| WWSC(A) | 45WH17 | 4100 | 500 |  | Charcoal | Montgomery 1979, Gaston 1975 |
| Beta-18050 | 45WH222 | 1190 | 95 |  | Charcoal | Wessen p.c. |
| Beta-22168 | 45WH223 | 6610 | 90 |  | Charcoal | McClure and Markos 1987 |
| Beta-33512 | 45WH224 | 1830 | 60 |  | Charcoal | Mierendorf 1993 |
| Beta-33514 | 45WH224 | 3980 | 80 |  | Charcoal | Mierendorf 1993 |
| Beta-33515 | 45WH224 | 4000 | 90 |  | Charcoal | Mierendorf 1993 |
| Beta-33516 | 45WH224 | 4090 | 90 |  | Charcoal | Mierendorf 1993 |
| Beta-33518 | 45WH224 | 7640 | 150 |  | Charcoal | Mierendorf 1993 |
| Beta-33519 | 45WH224 | 4590 | 80 |  | Charcoal | Mierendorf 1993 |
| WSU-3813 | 45WH224 | 4470 | 200 |  | Charcoal | Mierendorf 1989 |
| WSU-3184 | 45WH224 | 3814 | 130 |  | Charcoal | Mierendorf 1989 |
| Beta-33513 | 45WH224 | 3980 | 70 |  | Charcoal | Mierendorf 1993 |
| Beta-33521 | 45WH224 | 4790 | 70 |  | Charcoal | Mierendorf 1993 |
| Beta-33522 | 45WH224 | 290 | 70 |  | Charcoal | Mierendorf 1993 |
| Beta-27498 | 45WH224 | 2800 | 120 |  | Charcoal | Mierendorf 1993 |
| Beta-33520 | 45WH224 | 5030 | 100 |  | Charcoal | Mierendorf 1993 |
| Beta-33508 | 45WH228 | 310 | 70 |  | Charcoal | Mierendorf et al. 1998 |
| Beta-64820 | 45WH230 | 580 | 120 |  | Charcoal | Mierendorf et al. 1998 |
| Beta-40693 | 45WH237 | 230 | 80 |  | Charcoal | Mierendorf et al. 1998 |
| Beta-234920 | 45WH239 | 2460 | 40 |  | Charcoal | Bush 2008 |
| Beta-234921 | 45WH239 | 150 | 40 |  | Charcoal | Bush 2008 |
| Beta-234919 | 45WH239 | 2740 | 60 |  | Charcoal | Bush 2008 |
| Beta-53859 | 45WH239 | 1430 | 120 |  | Charcoal | Mierendorf et al 1998 |
| RL-149 | 45WH24 | 1580 | 120 |  | Charcoal | Grabert and Larsen 1975 |
| WWSC(B) | 45WH24 | 1500 | 120 |  | Charcoal | Gaston and Grabert 1975 |
| Beta-249112 | 45WH241 | 230 | 40 |  | Charcoal | Bush et. al. 2009 |
| Beta-249111 | 45WH241 | 190 | 40 |  | Charcoal | Bush et. al. 2009 |
| Beta-249118 | 45WH241 | 240 | 60 |  | Charcoal | Bush et. al. 2009 |
| Beta-249119 | 45WH241 | 220 | 40 |  | Charcoal | Bush et. al. 2009 |
| Beta-40695 | 45WH241 | 1890 | 90 |  | Charcoal | Mierendorf et al 1998 |
| Beta-40696 | 45WH241 | 1430 | 90 |  | Charcoal | Mierendorf et al 1998 |
| Beta-249113 | 45WH241 | 360 | 40 |  | Charcoal | Bush et. al. 2009 |
| Beta-249114 | 45WH241 | 230 | 40 |  | Charcoal | Bush et. al. 2009 |
| Beta-249115 | 45WH241 | 5020 | 40 |  | Charcoal | Bush et. al. 2009 |
| Beta-249116 | 45WH241 | 5030 | 40 |  | Charcoal | Bush et. al. 2009 |
| Beta-249110 | 45WH241 | 1370 | 40 |  | Charcoal | Bush et. al. 2009 |
| Beta-249117 | 45WH241 | 1750 | 40 |  | Charcoal | Bush et. al. 2009 |
| Beta-40697 | 45WH253 | 580 | 80 |  | Charcoal | Mierendorf et al 1998 |
| Beta-303702 | 45WH253 | 510 | 30 |  | Charcoal | Iversen et. al. 2012 |
| Beta-40698 | 45WH255 | 180 | 50 |  | Charcoal | Mierendorf et al 1998 |
| Beta-40699 | 45WH255 | 500 | 90 |  | Charcoal | Mierendorf et al 1998 |
| Beta-64821 | 45WH262 | 1120 | 80 |  | Charcoal | Mierendorf et al 1998 |
| Beta-65229 | 45WH262 | 310 | 70 |  | Charcoal | Mierendorf et al 1998 |
| Beta-64822 | 45WH262 | 1020 | 80 |  | Charcoal | Mierendorf et al 1998 |
| Beta-40700 | 45WH264 | 430 | 100 |  | Charcoal | Mierendorf et al 1998 |
| Beta-40701 | 45WH268 | 1760 | 80 |  | Charcoal | Mierendorf et al 1998 |
| Beta-40702 | 45WH283 | 1380 | 110 |  | Charcoal | Mierendorf et al 1998 |
| Beta-33509 | 45WH296 | 2670 | 70 |  | Charcoal | Mierendorf et al 1998 |
| Beta-64826 | 45WH300 | 1750 | 60 |  | Charcoal | Mierendorf et al 1998 |
| Beta-64824 | 45WH300 | 1650 | 70 |  | Charcoal | Mierendorf et al 1998 |
| Beta-64825 | 45WH300 | 1940 | 90 |  | Charcoal | Mierendorf et al 1998 |
| Beta-33510 | 45WH303 | 2530 | 150 |  | Charcoal | Mierendorf et al 1998 |
| Beta-220784 | 45WH303 | 3210 | 50 |  | Charcoal | Bush et al. 2007 |
| Beta-40703 | 45WH304 | 330 | 70 |  | Charcoal | Mierendorf et al 1998 |
| Beta-183744 | 45WH34 | 240 | 50 |  | Wood | Hutchings 2004 [Unpublished]; Gillis 2007 [Unpublished] |
| Beta-176487 | 45WH34 | 330 | 70 |  | Poplar branches | Hutchings 2004 [Unpublished]; Gillis 2007 [Unpublished] |
| Beta-183745 | 45WH34 | 420 | 50 |  | Wood | [Unpublished]; Gillis 2007 [Unpublished] |
| Beta-176491 | 45WH34 | 890 | 60 |  | Some mature Douglas fir wood and bark | [Unpublished]; Gillis 2007 [Unpublished] |
| RL-274 | 45WH34 | 1030 | 100 |  | Wood | [Unpublished]; Gillis 2007 [Unpublished] |
| Beta-176492 | 45WH34 | 1110 | 50 |  | Yew | [Unpublished]; Gillis 2007 [Unpublished] |
| RL-275 | 45WH34 | 1210 | 100 |  | Wood | [Unpublished]; Gillis 2007 [Unpublished] |
| Beta-192795 | 45WH34 | 4490 | 70 |  | Marine Shell | [Unpublished]; Gillis 2007 [Unpublished] |
| Beta-176490 | 45WH34 | 4010 | 40 |  | Mature red cedar | [Unpublished]; Gillis 2007 [Unpublished] |
| Beta-187079 | 45WH34 | 4850 | 80 |  | Marine Shell | [Unpublished]; Gillis 2007 [Unpublished] |
| Beta-192796 | 45WH34 | 4100 | 60 |  | Bone | [Unpublished]; Gillis 2007 [Unpublished] |
| Beta-192797 | 45WH34 | 4170 | 70 |  | Bone | [Unpublished]; Gillis 2007 [Unpublished] |
| RL-273 | 45WH34 | 4180 | 120 |  | Wood | [Unpublished]; Gillis 2007 [Unpublished] |
| Beta-187078 | 45WH34 | 4960 | 70 |  | Marine Shell | [Unpublished]; Gillis 2007 [Unpublished] |
| Beta-187080 | 45WH34 | 4970 | 80 |  | Marine Shell | [Unpublished]; Gillis 2007 [Unpublished] |
| Beta-176488 | 45WH34 | 4290 | 40 |  | Brown birch bark | [Unpublished]; Gillis 2007 [Unpublished] |
| Beta-176489 | 45WH34 | 4370 | 90 |  | (Old wood, Excluded) Poplar branches, hemlock or red cedar branches, mature conifer bark | [Unpublished]; Gillis 2007 [Unpublished] |
| Beta-298340 | 45WH4 | 370 | 30 | 420 | Marine Shell | Campbell 2013 (Database) |
| Beta-4104 | 45WH43 | 280 | 50 |  | Charcoal | Grabert and Griffin 1983 |
| Beta-307548 | 45WH47 | 2610 | 30 | 454 | Marine Shell | Palmer 2015 [Unpublished] |
| Beta-64828 | 45WH473 | 370 | 80 |  | Charcoal | Mierendorf et al 1998 |
| WSU-3411 | 45WH477 | 1350 | 60 |  | Bone | Mierendorf 2004 |
| Beta-73000 | 45WH477 | 1110 | 100 |  | Charcoal | Mierendorf 2004 |
| Beta-72999 | 45WH477 | 260 | 70 |  | Charcoal | Mierendorf 2004 |
| Beta-72998 | 45WH477 | 280 | 60 |  | Charcoal | Mierendorf 2004 |
| UW-311 | 45WH48 | 3495 | 125 |  | Charcoal | Gaston 1975, Bailey 1981 |
| N/A (A) | 45WH48 | 3700 | 200 |  | Charcoal | Gaston 1975, Bailey 1981 |
| Beta-293306 | 45WH521 | 1980 | 40 |  | Charcoal | Hovezak 2011 |
| Beta-285336 | 45WH525 | 1110 | 40 |  | Bone | Baldwin 2010 |
| Beta-285337 | 45WH525 | 1180 | 50 | 400 | Marine Shell | Baldwin 2010 |
| Beta-293546 | 45WH525 | 3680 | 50 |  | Marine Shell | Arthur et al. 2012 |
| D-AMS 001001 | 45WH525 | 1016 | 22 | 400 | Marine Shell | Iversen 2012 |
| D-AMS 001002 | 45WH525 | 1697 | 24 | 400 | Marine Shell | Iversen 2012 |
| D-AMS 001003 | 45WH525 | 1122 | 22 | 400 | Marine Shell | Iversen 2012 |
| D-AMS 001004 | 45WH525 | 1154 | 22 | 400 | Marine Shell | Iversen 2012 |
| Beta-225671 | 45WH526 | 610 | 50 |  | Charcoal | Baldwin et. al. 2007 |
| Beta-230664 | 45WH526 | 2570 | 50 | 400 | Marine Shell | Baldwin et. al. 2007 |
| Beta-234928 | 45WH526 | 1460 | 40 | 400 | Marine Shell | Baldwin et. al. 2007 |
| Beta-211703 | 45WH54 | 1030 | 40 | 390 | Marine Shell | Campbell 2013 (Database) |
| UW-331 | 45WH541 | 3495 | 125 |  | Charcoal | Gaston 1975 |
| Beta-311635 | 45WH541 | 2660 | 30 | 400 | Marine Shell | Hovezak 2014 |
| Beta-290596 | 45WH541 | 1220 | 40 | 400 | Marine Shell | Koziarski et al. n.d. |
| Beta-272637 | 45WH55 | 950 | 40 | 502 | Balanus, unidentifiable shell, Thais body, Mytilus | Lewis 2013 [Unpublished]; Pierce 2011 [Unpublished] |
| Beta-268774 | 45WH55 | 2370 | 40 | 390 | Prothaca | Lewis 2013 [Unpublished]; Pierce 2011 [Unpublished] |
| Beta-280399 | 45WH55 | 2910 | 50 | 464 | Marine Shell | Lewis 2013[Unpublished]; Pierce 2011 [Unpublished] |
| Beta-280400 | 45WH55 | 2510 | 40 |  | Bone | Lewis 2013 [Unpublished]; Pierce 2011 [Unpublished] |
| Beta-215323 | 45WH55 | 2880 | 50 | 390 | Myltitus and balanus | Lewis 2013 [Unpublished]; Pierce 2011 [Unpublished] |
| Beta-313564 | 45WH560 | 3780 | 30 |  | Bone | Mather 2012 |
| Beta-347784 | 45WH560 | 1570 | 30 | 400 | Marine Shell | Mather et al. 2016 |
| Beta-435204 | 45WH560 | 3360 | 30 |  | Charcoal | Mather et al. 2016 |
| Beta-440303 | 45WH560 | 3120 | 30 |  | Marine Shell | Mather et al. 2016 |
| Beta-175670 | 45WH564 | 1810 | 40 |  | Charcoal | Smith et al. 2004 |
| Beta-259404 | 45WH564 | 580 | 40 |  | Charcoal | Arthur et al. 2009 |
| Beta-259402 | 45WH564 | 100.6 | 0.05 |  | Charcoal | Arthur et al. 2009 |
| Beta-259403 | 45WH564 | 480 | 40 |  | Charcoal | Arthur et al. 2009 |
| Beta-283288 | 45WH564 | 2910 | 50 |  | Marine Shell | Baldwin 2010 |
| Beta-290597 | 45WH564 | 210 | 40 |  | Charcoal | Moreno et al. 2011 |
| Beta-290598 | 45WH564 | 1460 | 40 | 400 | Marine Shell | Moreno et al. 2011 |
| Beta-287565 | 45WH564 | 1520 | 40 | 400 | Marine Shell | Meidinger et al. 2011 |
| Beta-287564 | 45WH564 | 1280 | 40 |  | Bone | Meidinger et al. 2011 |
| Beta-287562 | 45WH564 | 1890 | 50 | 400 | Marine Shell | Meidinger et al. 2011 |
| Beta-287563 | 45WH564 | 1690 | 40 | 400 | Marine Shell | Meidinger et al. 2011 |
| Beta-300137 | 45WH564 | 2520 | 70 | 400 | Marine Shell | Arthur et al. 2011 |
| Beta-301250 | 45WH564 | 2210 | 50 | 400 | Marine Shell | Arthur et al. 2011 |
| Beta-301251 | 45WH564 | 2400 | 60 | 400 | Marine Shell | Arthur et al. 2011 |
| Beta-301252 | 45WH564 | 2220 | 60 | 400 | Marine Shell | Arthur et al. 2011 |
| Beta-301436 | 45WH564 | 1810 | 60 | 400 | Marine Shell | Moreno et al. 2011 |
| Beta-301437 | 45WH564 | 1720 | 60 | 400 | Marine Shell | Moreno et al. 2011 |
| Beta-286447 | 45WH564 | 1360 | 50 | 400 | Marine Shell | Baldwin et al. 2010 |
| Beta-286448 | 45WH564 | 790 | 50 |  | Charcoal | Baldwin et al. 2010 |
| Beta-298058 | 45WH57 | 7030 | 40 |  | Charcoal | Koziarski et al. 2011 |
| Beta-213173 | 45WH67 | 960 | 40 |  | Charcoal | Shantry et al. 2012 |
| Beta-427721 | 45WH67 | 1010 | 30 |  | Bone | Baldwin 2016 |
| Beta-427722 | 45WH67 | 1130 | 30 | 400 | Marine Shell | Baldwin 2016 |
| Beta-290203 | 45WH72 | 3830 | 50 |  | Marine Shell | Chambers et al. 2011 |
| Beta-290205 | 45WH72 | 3750 | 40 |  | Marine Shell | Chambers et al. 2011 |
| Beta-290204 | 45WH72 | 2830 | 40 |  | Bone | Chambers et al. 2011 |
| Beta-209214 | 45WH726 | 950 | 40 |  | Charcoal | Shong et al. 2005 |
| Beta-227261 | 45WH735 | 1050 | 60 |  | Marine Shell | Wessen 2007 |
| Beta-211705 | 45WH758 | 1090 | 40 | 390 | Marine Shell | Campbell 2013 (Database) |
| Gx-32437 | 45WH758 | 1740 | 50 | 67 | Marine Shell | Campbell 2013 (Database) |
| Beta-227507 | 45WH758 | 1350 | 40 | 390 | Marine Shell | Campbell 2013 (Database) |
| Beta-211704 | 45WH763 | 2730 | 40 | 390 | Marine Shell | Campbell 2013 (Database) |
| N/A (B) | 45WH764 | 780 | 40 |  | Marine Shell | Elder and Smart 2007 |
| UW-343 | 45WH9 | 848 | 108 |  | Charcoal | Gaston and Grabert 1975 |
| #81 | 45WH9 | 1285 | 88 |  | Charcoal | Gaston and Grabert 1975 |
| #82 | 45WH9 | 3125 | 90 |  | Charcoal | Montgomery and Grabert 1977 |
| Beta-278908 | 45WH9 | 520 | 40 |  | Charcoal | Baldwin et al. 2012 |
| Beta-278905 | 45WH9 | 1490 | 40 | 400 | Marine Shell | Baldwin et al. 2012 |
| Beta-293889 | 45WH9 | 540 | 30 |  | Charcoal | Baldwin et al. 2012 |
| Beta-278910 | 45WH9 | 1710 | 40 | 400 | Marine Shell | Baldwin et al. 2012 |
| Beta-278909 | 45WH9 | 950 | 40 |  | Bone | Baldwin et al. 2012 |
| Beta-278906 | 45WH9 | 710 | 40 |  | Charcoal | Baldwin et al. 2012 |
| Beta-278907 | 45WH9 | 690 | 98 |  | Charcoal | Baldwin et al. 2012 |
| Beta-351366 | 45WH947 | 1890 | 30 |  | Charcoal | Stegner et. al. 2016 |
| Beta-371977 | 45WH947 | 1970 | 30 |  | Charcoal | Stegner et. al. 2016 |
| Beta-209901 | WWU01 | 1080 | 79 | 390 | Marine Shell | Campbell 2013 (Database) |
| Beta-209902 | WWU99-01 | 910 | 60 | 390 | Marine Shell | Campbell 2013 (Database) |

**Supplemental References (References in article are excluded)**

Arthur EP, Baldwin GL. Archaeological Investigation and Monitoring at 81 Bay View Drive, TPN4153355333159. 2009.

Arthur EP, Baldwin GL. Emergency Archaeological Monitoring and Excavation within 45WH564 for Completion of Residential Construction at 2146 Fir Street, Maple Beach, Point Roberts, Whatcom County, Washington. 2011.

Arthur EP. Additional Testing and Archaeological Monitoring of Septic System Replacement at 1831 Edwards Drive (TPN 405311 034372. Washington: South Beach, Point Roberts, Whatcom County; 2012.

Arthur EP, Mather CA. Results of Archaeological Monitoring and Data Recovery at 164 Bunny Lane. Washington: Orcas Island; 2014.

Baldwin GL. Letter to Mike Fernandes Regarding Friday Harbor Seawall Project Monitoring. 2007.

Baldwin GL, Arthur E, Brown G, Kaiser C. Archaeological Damage Assessment of 45WH526 Marietta. Washington: Whatcom County; 2007.

Baldwin, GL. Watrous JB. Archaeological Assessment of 2159 Alder Street (TPN 415335 520205), Point Roberts, Whatcom County, Washington 98281. 2010

Baldwin GL, O’Brien M, Mather CA. Archaeological Assessment of the Proposed Septic System Replacement at 1831 Edwards Drive (TPN 405311 034372. Washington: South Beach, Point Roberts, Whatcom County; 2010.

Baldwin GL, Meidinger BN, Arthur EP. Birch Bay Water and Sewer District’s Force Main Replacement Project: Monitoring and Excavation at Archaeological Site 45WH9. Washington: Birch Bay State Park, Whatcom County; 2012.

Baldwin GL. Monitoring Report for the Proposed Birch Bay Park. Birch Bay, Whatcom County, Washington; 2016.

Bard JC, Ballantyne R, McClintock RD, Sharpe JJ, Cheung J, Gleason eArchaeological Data Recovery at 45-SJ-165 and 45-SJ-169 Decatur Island, San Juan County, Washington for the Anacortes Fiber Project. 2007

Benson JR. Archaeological Testing at 45-SJ-274. Spencer Spit State Park. San Juan County, Washington. Submitted to the Washington State Parks and Recreation Commission. Office of Public Archaeology Institute for Environmental Studies University of Washington; 1981.

Blodgett ME. Cherry Point: A Strait of Georgia Fishing Station. 1976.

Onat OB, Bennett LA, Hollenbeck JL. In: Cultural Resources Survey Skagit River Levees and Channel Improvement Project Below Mount Vernon. Washington; 1980.

Bovy K, Phillips L, Stein J. Watmough Bay Site Stabilization Project 45-SJ-280 Descriptive Preliminary Report. 2005.

Bush KR, Ferry JC, Elder JT. In: Final Report: Ross Lake Archaeological Data Recovery Project: 45WH234 and 45WH303 North Cascades NPS Complex Ross Lake. Washington; 2007.

Bush KR, Smart T, Meidinger BN. In: Final Report: Ross Lake Archaeological Data Recovery Project: 45WH239 North Cascades NPS Complex Ross Lake. Washington; 2008.

Bush KR, Meidinger BN, Peebles A. In: Final Report: Ross Lake Archaeological Data Recovery Project: 45WH241 North Cascades NPS Complex Ross Lake. Washington; 2009.

Bush KR, Smart TS, Roland JM. In: Archaeological Investigation and Monitoring Report: Fisher Slough Freshwater Tidal Marsh Restoration Project, Skagit County. Washington; 2011.

Chambers JL, Baldwin GL. Archaeological Investigations at 45WH72. Washington: Birch Bay State Park, Whatcom County; 2011.

Dubeau MA. Late-Holocene Mammal Use in the Salish Sea: A Case Study from the Cherry Point Site (45WH1). Northwestern Washington; [Unpublished MA Thesis]. Department of Anthropology Western Washington University 2012.

Elder T, Smart T. Archaeological Site Inventory Form 45WH764. In: On File with the Department of Archaeology and Historic Preservation. 2007.

Gaston JL. The Extension of the Fraser Delta Cultural Sequence into Northwest Washington. 1975.

Gaston JL, Grabert GF. Salvage Archaeology at Birch Bay Washington. A Report of Investigations Conducted in. 1975.

Gillis NA. An Investigation of Charles Culture Housepit Deposits at the Ferndale Site (45WH34). [Unpublished MA Thesis]. Department of Anthropology Western Washington University 2007.

Grabert GF, Larsen CE. Marine Transgressions and Cultural Adaptation: Preliminary Tests of an Environmental Model. In: Mouton WF, editor. Prehistoric Maritime Adaptations of the Circumpolar Zone. 1975.

Grabert GF, Griffin G. Archaeological Investigations on the Lummi Indian Reservation, Washington. Department of Anthropology Western Washington University; 1983.

Hovezak MJ. Excavation of a Pre-Contact Fire Hearth Site at 45WH521. Washington: Lummi Island, Whatcom County; 2011.

Hovezak MJ. Archaeological Site Inventory Form 45WH541. On File with the Department of Archaeology and Historic Preservation. 2014.

Hutchings RM. Mid-Holocene River Development and South-Central Pacific Northwest Coast Prehistory: Geoarchaeology of the Ferndale Site (45WH34. Nooksack River, Washington; 2004.

Iversen DR. Archaeological Damage Assessment of 45WH525 at 725 South Beach Road, Point Roberts. Washington: Whatcom County; 2012.

Iversen D, R. OTO, Becker MS. Data Recovery at 45WH253. In: Ross Lake Reservoir, North Cascades National Park, Whatcom County, Washington On File with the Department of Archaeology and Historic Preservation. 2012.

Kenady S, Schalk R, Wolverton M, Weiser A, Stenholm N. Feature Excavation and Analysis At 45SJ53. San Juan County, Washington. Center for Northwest Anthropology Washington State University.; 2004.

Kenady SM. Archaeological Analysis of a Utilities Upgrade Project in Mackaye Harbor, Lopez Island: 45SJ186. On File with the Department of Archaeology and Historic Preservation. 1996.

Kenady SM. Archaeological Site Inventory Form 45SJ414. On File with the Department of Archaeology and Historic Preservation. 2002.

Kenady SM, Schalk R, Wolverton M, Weiser A, Stenholm N. Feature Excavation and Analysis At 45SJ53. San Juan County, Washington; 2004.

Kidd. Robert S. A Synthesis of Western Washington Prehistory from the Perspective of Three Occupation Sites. University of Washington; 1964

Kidd. Robert S. A Final Report on Archaeological Investigation conducted during the Summer of 1960 at Site 45SJI05, Sucia Island, Northwestern Washington, under the Sponsorship of the Washington State Parks and Recreation Commission; 1965

Kopperl RE. Washington State Archaeological Site Inventory Form, 45SK258. On File with the Washington State Department of Archaeology and Historic Preservation. Olympia; 2011.

Koziarski R, Meidinger B, Baldwin GL. Archaeological Testing of 45WH57, at the Wilder Industrial Park Location. Ferndale, Whatcom County, Washington; 2011.

Koziarski R, Baldwin G. Archaeological Monitoring and Mitigation of the Petrie Shell Midden (45WH541) at 5925 Normar Place. Blaine, Whatcom County, Washington: Birch Point; 2011.

Larson LL, Lewarch DE. Report on Testing of 45SK200 Washington: Near Bacon Creek, Skagit County; 1989.

Lewis I. Chasing Clusters: An Analysis of Activity Areas to Determine Site Type at the Locarno Beach Phase (3500-2400 BP) Site 45WH55, Chuckanut Bay, Washington. [Unpublished MA Thesis]. Department of Anthropology Western Washington University 2013.

Mather CA. Locarno Beach Period (3500-2400 BP) Settlement and Subsistence in the Gulf of Georgia Region: A Case Study from Site 45SK46, Deception Pass. Washington.[Unpublished MA Thesis]. Department of Anthropology Western Washington University; 2009.

Mather CA. Archaeological Monitoring of Residential Development at 601 Marine Drive (TPN 405309 501579. Washington: Point Roberts, Whatcom County; 2012.

Mather CA, Arthur E, . Results of Archaeological Monitoring and Data Recovery at 581 Marine Drive. Washington: Point Roberts, Whatcom County; 2016.

McClure RH, Markos JA. Archaeological Testing and Evaluation of the Damfino Lakes Site (45-WH-223) Mt. Baker-Snoqualmie National Forest; 1987.

Meidinger BN, Baldwin GL, Moreno MA. Archaeological Monitoring of Septic Installation and Matrix Sample Results from 45WH564 at 2154 Elm Street. Washington: Point Roberts, Whatcom County; 2011.

Mierendorf RR. People of the North Cascades. Washington: National Park Service Pacific Northwest Region Seattle; 1986.

Mierendorf RR. Archaeology of the Desolation Chert Quarry (45WH224). 1989.

Mierendorf RR. Chert Procurement in the Upper Skagit River Valley of the Northern Cascade Range. Washington: Ross Lake National Recreation Area; 1993.

Mierendorf RR, Harry DJ, Sullivan GM, Forrest JH, Harsha JO, Kennady JG III, et al. In: An Archaeological Site Survey and Evaluation in the Upper Skagit River Valley, Whatcom County. Washington; 1998.

Mierendorf RR. Cultural Resources Survey of SR-20 at Falls and Afternoon Creeks, Ross Lake National Recreation Area, Whatcom County, Washington. In: Prepared by Resource Management Division North Cascades National Park Service Complex Sedro Woolley. Washington; 2004.

Montgomery KR, Grabert GF. Birch Bay State Park II: Archaeological Investigations on Newly Acquired Property at Birch Bay State Park April. 1977.

Montgomery KR. Prehistoric Settlements of Sumas Valley, Washington. [Unpublished MA Thesis]. Department of Anthropology Western Washington University;1979.

Moreno MA, Meidinger BN, Baldwin GL. Archaeological Monitoring of Septic Installation and Mitigation Results from 45WH564 at 85 Bayview Drive. Washington: Point Roberts, Whatcom County; 2011.

Nelson MA, Schalk RF, Trost T, Wolverton M, Boersema J. Archaeological Testing at the Weaverling Spit, Site 45SK43. Washington: Skagit County; 2010.

Nelson MA, Trost T, Kenady SM. Archaeological Monitoring at Site 45SJ200. San Juan County, Washington: Lopez Island; 2011.

Nelson MA, Wolverton MJ, Trost T, Helzer M. Archaeological Monitoring and Excavations in the Waterfront Area of the Snug Harbor Resort, 45SJ13. San Juan County; 2014.

Palmer J. A Fresh Look at an Old Artifact: A New Interpretation of Edged Cobbles at Cherry Point. Department of Anthropology, Western Washington University. [Unpublished MA Thesis]. Department of Anthropology Western Washington University; 2015.

Pierce SD. Bivalve Growth Stages as a Measure of Harvesting Intensity: Application on the Southern Northwest Coast. [Unpublished MA Thesis]. Department of Anthropology Western Washington University; 2011.

Robinson SW, Thompson G. Radiocarbon Corrections for Marine Shell Dates with Application to Southern Pacific Northwest Coast Prehistory. 1980.

Rorabaugh AN. Barbed Bone and Antler Technologies: Cultural Transmission and Variation in the Gulf of Georgia. Northwest North America; [Unpublished MA Thesis]. Department of Anthropology Western Washington University; 2009.

Shantry K, Kopperl R, Shong M. Data Recovery Excavations at 45WH67, Sandcastle Condominium Locality. Birch Bay, Whatcom County, Washington; 2012.

Shong MV, Stevenson AE, Miss CJ. Report of Cultural Resources Monitoring at the Coast Millennium Trail Squalicum Connector, Segment A and Archaeological Testing at Site 45WH726, Whatcom County; 2005.

Smith R, Anderson S, Campbell S, Johnnie AS. The Results of the Garth Road Recovery Project Conducted at the Meikle Property, 2168 Garth Road, Point Roberts. Washington. 2004.

Stegner M, McDaniel S. Relocation of Precontact Artifacts and Materials Encountered During Archaeological Monitoring of the BP Refinery Rail Logistics Project. 2016.

Stein JK. Interpreting the Stratigraphy of Northwest Shell Middens. Willig JA, editor. Geoarchaeology of the Northwest: Recent Applications and Contributions. 1984;26–34. Stein JK. Monitoring Report for the Cattle Point Site, 2004 Excavation of Soil Pit 45SJ01. University of Washington; 2005.

Stein JK, Taylor AK, Jolivette SAE. Archaeological Excavations at 45-SJ-200. Burke Museum of Natural History and Culture University of Washington; 2010.

Taber E. Changes in Intertidal Mollusk Harvesting at Cherry Point Archaeological Site (45WH1). [Unpublished MA Thesis]. Department of Anthropology Western Washington University 2010.

Taylor AK. Creating and Transcending Territorial Boundaries in Late Holocene Pacific Coast Communities [Unpublished PhD Dissertation.]. Department of Anthropology, University of Washington; 2012.

Tierney A. Walking with Wapiti: Measuring Late Holocene Climatic Variability Through Cervus elaphus Abundance and Stable Isotope Analysis in the Gulf of Georgia Region. [Unpublished MA Thesis]. Department of Anthropology Western Washington University 2012.

Thompson EN. Historic Resource Study San Juan Island National Historical Park Washington. Denver Service Center; 1972.

Thompson G. Prehistoric Settlement Changes in the Southern Northwest Coast: A Functional Approach. 1978.

Trost T, Kenady SM, Nelson MA. Data Recovery Excavations at Site 45JS200 Lopez Island. San Juan County, Washington; 2010.

Walker SL, lves RS, Luttrell CT, Lyman RL, Wigen RJ, Stein JK, Anderson, P, O’Neal MA, Hughes RE Archaeological Investigations at Sites 45SJ165 and 45SJ169, Decatur Island, San Juan County, Washington; 2003

Walker SL, Thomas G, Weiser A, Ives R, Smith T. In: Cultural Resource Monitoring and Data Recovery Excavations at Site 45SK139 for the Marblemount Water System Project, Skagit County. Washington; 2009.

Wessen GC. Archaeological Site Testing Activities at 45WH735. Bellingham, Whatcom County, Washington; 2007.

Wessen GC. Archaeological Data Recovery Activities at the Tompkins Cabin Site (45SJ540. Washington: Madrona Point, Orcas Island; 2013.

Wessen GC, Morris J. Archaeological Testing at Smallpox Bay (45-SJ-11) San Juan Island, Washington and An Ethnographic Enquiry into the Historic Native American Use of the Smallpox Bay Area of San Juan Island. A Report Prepared for the San Juan County Parks Board by Wessen & Associates, Inc. Washington; 1988.
