# Supplementary material for: Assessing population dynamics in the Central Salish Sea, Pacific Northwest Coast of North America
Source: PLoS One. 2023 Aug 3;18(8):e0285021. doi: 10.1371/journal.pone.0285021 (PMC10399822; doi:10.1371/journal.pone.0285021)
Supplement: Attachment — Submitted filename: Response to Reviewers.doc [file pone.0285021.s002.doc]

Response to editor comments:

Both reviewers indicate that your work has promise to be an important contribution to understanding demographic trends in your study area. However, they also indicate the need for fairly substantial revisions and additions. Reviewer 1 indicates the need for much more contextual information. I agree. When I first read your manuscript, I thought it read like a section/chapter in a much larger report rather than a stand alone journal article. Reviewer 2 indicates the need for corrections in your discussion of kernel density estimates. As implemented in OxCal KDEs are not strictly Bayesian: "Because the KDE method is in itself frequentist, this is not a purely Bayesian approach" (Bronk Ramsey 2017:1819). To better place KDEs in context, I suggest that you read: Crema, Enrico R. "Statistical inference of prehistoric demography from frequency distributions of radiocarbon dates: a review and a guide for the perplexed." Journal of Archaeological Method and Theory (2022): 1-32. As Reviewer 2 indicates there are Bayesian approaches to analyzing compiled radiocarbon dates for demographic modeling. Baydem is one possibility, but you do not need to do so for this paper. You do need to correct the sentence on assumptions about underlying distributions. Finally the paper needs a careful proofread. As both reviewers indicate there are partial sentences throughout.

Additional contextual information has been added to the article to address that issue and provide more concise hypotheses to test against. Reviewer #2’s option 1 was selected for the edits and in areas for future work that has been added as a future option. A settlement pattern analysis (as suggested by Reviewer #1) and Baydem approach on specific sites will be a next step.

Additional proofreading has been completed for the article. I hope that this revised version of the manuscript addresses the concerns raised and the following includes how each issue has been addressed.

1. Please ensure that your manuscript meets PLOS ONE's style requirements, including those for file naming.

Manuscript has been adjusted to meet style requirements including file naming.

2. In your manuscript, please provide additional information regarding the specimens used in your study. Ensure that you have reported specimen numbers and complete repository information, including museum name and geographic location.

Radiocarbon date laboratory numbers have been provided. Citations in the supplemental data provide the original reports and additional supplemental information. In many cases dates taken in CRM contexts were sent to radiocarbon labs with no remaining sample for a repository.

If permits were required, please ensure that you have provided details for all permits that were obtained, including the full name of the issuing authority, and add the following statement:

"All necessary permits were obtained for the described study, which complied with all relevant regulations."

If no permits were required, please include the following statement:

"No permits were required for the described study, which complied with all relevant regulations."

No permit text has been added.

3. We note that you have referenced Daniels (2009), Graesch (2006), Palmer (2015), Taylor (2012), Tierney (2012) which has currently not yet been accepted for publication. Please remove this from your References and amend this to state in the body of your manuscript: (ie Daniels et al. [Unpublished]) as detailed online in our guide for authors

These are MA theses and dissertations, have updated references to style guide and added unpublished.

4. Please ensure that you refer to Figure 10 in your text as, if accepted, production will need this reference to link the reader to the figure.

Figure 10 has been referred to in text.

5. We note that Figures 1,2,3,7, and 11 in your submission contain map images which may be copyrighted. All PLOS content is published under the Creative Commons Attribution License (CC BY 4.0), which means that the manuscript, images, and Supporting Information files will be freely available online, and any third party is permitted to access, download, copy, distribute, and use these materials in any way, even commercially, with proper attribution. For these reasons, we cannot publish previously copyrighted maps or satellite images created using proprietary data, such as Google software (Google Maps, Street View, and Earth). For more information, see our copyright guidelines: http://journals.plos.org/plosone/s/licenses-and-copyright.

Basemaps have been adjusted to use CC BY 4.0 compliant layers. Specifically Natural Earth vector data:

https://www.naturalearthdata.com/downloads/10m-physical-vectors/

6. Please include captions for your Supporting Information files at the end of your manuscript, and update any in-text citations to match accordingly. Please see our Supporting Information guidelines for more information: http://journals.plos.org/plosone/s/supporting-information.

Captions have been provided.

Response to Reviewer #1:

Reviewer #1: The author has compiled 538 radiocarbon dates from 142 archaeological sites across northwestern Washington State. The dates are grouped into three regions in order to compare demographic trends between them and also with other demographic models existing to the north, in British Columbia, Canada. Utilizing and comparing marine shell dates was also an objective of this study.

The compilation and analysis of these radiocarbon dates has the potential to be a significant contribution, but this study, as is, provides only a cursory and uncritical summary of the data, description of the results, and discussion of demographic trends. More contextual information is necessary for anything beyond the most basic modelling of demography. As it is, a reader would have a challenging time trying to evaluate the appropriateness of the research questions or the validity of the interpretations because no background information in offered on the nature of the c14 dated sites, or the known settlement history of the region. Readers unfamiliar with the region would reasonably wonder how the analysis of the compiled c14 dates relates to earlier research in the region, and advances understanding of key anthropological or historical questions.

A background section has been added to specifically note research questions in the region and their role in broader anthropological discussions. Additionally, it emphasizes that this paper is intended to provide a data synthesis similar to that done in BC and notes a model in BC to contrast large scale patterns with (Clark 2013).

Another concern with this study is that there is no effort to look at the settlement patterning over time, to detect if the types of sites or the distribution of sites changed in relation to one another, the environment, new technologies and settlement forms/size, or demographic trends. There is no discussion of factors that might help to explain some of the modeled ups and down in the KDE.

The background section should help address this concern. However settlement pattern analysis is not the focus of this particular paper nor is an analysis of site size and scope. An discussion of residential site sizes is in Rorabaugh 2017. Stein (2003) provides a seminal discussion of shell midden site formation that can also make behavioral interpretations based on site size alone problematic.

Additional language has been added to link the results to the background section but also to highlight that the processes are not simply tied to population pressure or resource intensification.

I am not qualified to determine if the marine reservoir corrections have been applied appropriately, but more details would be helpful, and critical analysis of the methods should also be provided considering the ongoing uncertainty and regional variability with using shell dates.

Daniels (2009), Osterkamp et al. (2014), and Deo et al. (2004) provide more in depth discussions of marine shell calibrations. Which is beyond the scope of this paper beyond emphasizing that the error term introduction from these dates is appropriate for meso scale analyses.

The KDE seem to indicate some interesting trends that are generally consistent with those of other studies, but again, there is not sufficiently detailed results to know what the significance of this might be. Where the models indicate differing trends, there is also not discussion of why this might be.

The significance is that the US-Canadian border is an artificial barrier dividing academic research of a cultural area with shared history. Additional sentences have been added pertaining to an alternative model (Clark 2013) and that the dataset does not correspond with the idea of a core Fraser Delta and periphery comprised of the sample.

This manuscript contains many sentence fragments (including the first line of the introduction) and some repetition. Other sections are too brief to be clear to the reader. A background section that might provide useful context for interpretations and discussion is missing.

A new background section has been added that should address these concerns. The manuscript has been gone through for sentence clarity.

Altogether, I'd like to see a research paper along these lines published, but would expect it to be thoroughly re-worked, from the intro to the conclusion.

Based on the comments it is unclear what another paper would look like that they are requesting except for a quite different settlement pattern analysis which is not the thrust of this study.

However, a background section and conclusions highlighting the specific findings situated in that background context have been added which should address these concerns within the scope of the aim of this study.

From reviewer #2 and #1s comments some additional areas for future work have been added which may be the paper that reviewer #1 may be envisioning but, again, is beyond the scope of this particular study. The settlement pattern analyses requested would be a different undertaking involving assessing spatial data quality from various sources but I would point the reviewer to the Taylor et al. article and Rorabaugh 2017 chapter 2 for discussions on household change through time and San Juan island site settlement pattern change through time.

Response to Reviewer #2

Reviewer #2: This paper updates Taylor et al.'s 2011 paper, exploring population growth dynamics of the Pacific Northwest using the temporal distributions of radiocarbon datasets as proxies for the increasing and decreasing abundance of the archaeological record over time, i.e. the so-called "dates as data" approach. The present manuscript expands on Taylor et al.'s previous work in two noteworthy ways: first, it expands the geographic scope and consequently the sample size of the radiocarbon datasets used by Taylor et al., including adding inland sites from Washington State. Second, it replaces the summed probability distribution (SPD) approach used by Taylor et al. for describing temporal distributions of data points with a kernel density estimation (KDE) approach. Specifically, the author applies the KDE functions introduced to OxCal by Bronk Ramsey (2017), which have the appeal of being (relatively) easily applied by OxCal users without requiring any extensive programming on the user's part.

In general, I think this manuscript makes a novel and nontrivial contribution to our demographic-archaeological knowledge of the Pacific Northwest, and I do hope that it will be published for this reason. I also think that OxCal's KDE functionality supersedes both the SPD approach and other "composite kernel density estimation" alternatives suggested by others reviewed in the submission (e.g. Brown 2017). There is one alternative approach on offer---finite mixture modeling---that is founded on even strong theoretical and methodological principles than OxCal's KDE approach, which I discuss further below, but I will leave it to the author to decide whether the added effort to pursue finite mixture models over a KDE models is worth it; OxCal's KDE functions are still great tools.

Agreed with finite mixing model and would be interested in working with Reviewer #2 on such an approach as a future step.

One thing I would strongly recommend is that the author give the manuscript a new round of revisions with fresh eyes especially for broken grammar. Most of the paper is clearly written, but I did encounter a handful of sentence fragments, primarily sentence subjects lacking their predicates. Make sure every sentence ends.

Manuscript has been checked for incomplete sentences and a pass for incomplete grammar.

Grammar aside, I do have one important critique of the manuscript that I think will need to be addressed before it is published: the author's characterization of kernel density estimation (KDE) as a Bayesian method is incorrect. In fact, KDE is not readily available to standard likelihood based approaches to model-fitting---whether maximum likelihood estimation (MLE; Silverman 1986: 25-26, 110-119; Simonoff 1996: 64-70) or Bayesian inference (Bouveyron et al. 2019: 92-93)---because tuning the KDE model's single free parameter ("bandwidth") in a likelihood-based way leads to the statistical problem of degeneracy: the likelihood function approaches infinity as the strictly positive bandwidth parameter approaches 0. In the case of KDE and other finite mixture models (FMMs), this degeneracy problem sometimes goes under the label "the collapsing variance problem" (Murphy 2012: 357-359), and it is unavoidable in the case of KDE. While it is true in general that OxCal comprises a diverse set of tools for radiocarbon data analysis that are Bayesian in a strict sense, this is not the case for OxCal's recently added KDE operations, a fact about which Bronk Ramsey (2017) is quite explicit. Instead, in order to operationalize KDE, OxCal's almost-but-not-quite-Bayesian approach replaces the proper likelihood function required by Bayes' Rule with a leave-one-out predictive probability function (Bronk Ramsey 2017: Eqs. 5 and 9). (A similar approach has been applied to MLE approaches to KDE; Silverman 1986: 52-55.) On one hand, this substitution "works" because the out-of-sample predictive probability function behaves in a manner similar to likelihood functions, increasing and decreasing over the parameter space depending on how suitable different parameter values are for the data under analysis. On the other hand, these out-of-sample predictive probabilities still should not be mistaken for likelihoods in the strict sense, so their substitution is of questionable theoretical merit. In my opinion, this choice is especially problematic for Bayesian inference because this substitution sacrifices the logical axioms that give this inferential framework, especially Bayes' Rule, its principled foundations. Nevertheless, a certain amount of pragmatic justification does exist for their use as a hack when proper likelihoods just won't work (Silverman 1986: 52-55), so I wouldn't insist that we abandon OxCal's KDE functions altogether. Even so, they are not Bayesian.

In any case, the author will need to make one of two changes to address and resolve this issue:

(1) The simpler solution would be editorial: remove or qualify any characterization of KDE as Bayesian, including in the paper title, key words, abstract, and main body of the text. This would require no new analysis on the author's part but merely a more accurate characterization of what the author has already done.

Approach #1 was taken, and a more accurate characterization of the work done has been included. Approach #2 would be an excellent approach to start with a smaller portion of the dataset such as 45WH1 Cherry Point or the Woodstock Farm site where there are strong

(2) Alternatively, if the author really wishes to do something that is truly Bayesian, he should consider a new round of analysis, replacing his KDE models with FMMs that can be approached in a Bayesian way. As it turns out, KDE models are an extreme case of FMMs, in which there are as many mixture components as there are data points and in which all kernels or mixture components share the same bandwidth. However, unlike KDE models specifically, FMMs more generally *are* available to Bayesian inference in the strict sense, particularly if there are fewer kernels/mixture components than there are data points. The collapsing variance problem mentioned earlier is still a possibility particularly for FMMs with large numbers of mixture components, but this problem can be mitigated in a Bayesian framework using regularizing priors (Fraley and Raftery 2007; Bouveyron 2019: 92-95) and/or posterior maximization rather than likelihood maximization (Murphy 2012: 357-359). Additionally, using FMMs with relatively few mixture components usually out-performs KDEs when applied as density estimates to the same data, particularly if different kernels/mixture components are allowed to have different bandwidths (Fraley and Raftery 2002; Gelman et al. 2013: 535). Unfortunately, OxCal has not yet implemented functions for finite mixture modeling. To my knowledge, the only prepackaged program capable of fitting such models is the 'baydem' package for R described by Price et al. (2021). If the author wishes to apply a truly Bayesian approach to the task at hand and is knowledgeable about how to use R (including loading packages from Github), I don't see any alternative at present to using 'baydem'.

One further, minor note: the author makes the assertion that "KDE is a non-parametric method and does not require assumptions of a particular underlying distribution, unlike parametric Bayesian approaches or summed probability distributions." Two thirds of this statement are true, but it is incorrect to say that summed probability distributions require assumptions of a particular underlying distribution. When producing SPDs, one can but need not make any such assumptions, and in fact in most cases users of SPDs do not do so.

The sentence has been corrected to remove the SPD portion to be accurate.

Overall, the provided edits to the manuscript should fully address Reviewer #2's concerns and should address comments by Reviewer #1 that were within the intended scope of this article. Additionally the editorial edits should be addressed with this version of the manuscript.

Sincerely,

Adam N. Rorabaugh, Ph.D.

Director, Heritage Resource Management Program

Department of Archaeology, Simon Fraser University
