# Supplementary material for: Assessing population dynamics in the Central Salish Sea, Pacific Northwest Coast of North America
Source: PLoS One. 2023 Aug 3;18(8):e0285021. doi: 10.1371/journal.pone.0285021 (PMC10399822; doi:10.1371/journal.pone.0285021)
Supplement: Attachment — Submitted filename: Response to Reviewers.doc [file pone.0285021.s003.doc]

Response to Reviewers:

1. If the authors have adequately addressed your comments raised in a previous round of review and you feel that this manuscript is now acceptable for publication, you may indicate that here to bypass the “Comments to the Author” section, enter your conflict of interest statement in the “Confidential to Editor” section, and submit your "Accept" recommendation.

Reviewer #1: (No Response)

Reviewer #2: (No Response)

2. Is the manuscript technically sound, and do the data support the conclusions?

The manuscript must describe a technically sound piece of scientific research with data that supports the conclusions. Experiments must have been conducted rigorously, with appropriate controls, replication, and sample sizes. The conclusions must be drawn appropriately based on the data presented.

Reviewer #1: Partly

Additional edits have been made to better connect the requested background sections and discussion as working hypotheses of the data including connecting the ethnographic discussions more explicitly with population dynamics and the addition of corroborating oral tradition. However, as reviewer #2 is a subject matter expert regarding the methods, if there are technical issues with the data, statistics, and presentation, I defer to them on those matters

Reviewer #2: Yes

3. Has the statistical analysis been performed appropriately and rigorously?

Reviewer #1: Yes

Reviewer #2: Yes

4. Have the authors made all data underlying the findings in their manuscript fully available?

The PLOS Data policy requires authors to make all data underlying the findings described in their manuscript fully available without restriction, with rare exception (please refer to the Data Availability Statement in the manuscript PDF file). The data should be provided as part of the manuscript or its supporting information, or deposited to a public repository. For example, in addition to summary statistics, the data points behind means, medians and variance measures should be available. If there are restrictions on publicly sharing data—e.g. participant privacy or use of data from a third party—those must be specified.

Reviewer #1: Yes

Reviewer #2: (No Response)

5. Is the manuscript presented in an intelligible fashion and written in standard English?

PLOS ONE does not copyedit accepted manuscripts, so the language in submitted articles must be clear, correct, and unambiguous. Any typographical or grammatical errors should be corrected at revision, so please note any specific errors here.

Reviewer #1: Yes

Reviewer #2: No

All suggested edits by reviewer #2 have been incorporated and additional copy editing for sections altered to address reviewer #1.

6. Review Comments to the Author

Please use the space provided to explain your answers to the questions above. You may also include additional comments for the author, including concerns about dual publication, research ethics, or publication ethics. (Please upload your review as an attachment if it exceeds 20,000 characters)

Reviewer #1: The author’s removal of the term “settlement” from the title of the manuscript helps to clarify the focus of this paper on population, but the rest of the edits do not address the principal issues that I identified.

As originally noted, the manuscript (from the first paragraph of the introduction) pre-supposes that readers will have an understanding of the paleo demography of the San Juan Islands and Salish Sea. I think that more work is required to demonstrate how this study contributes to the study of population dynamics in the region.

The paper now explicitly defines the Salish Sea, and I have included that this is the first examination of dates for this region beyond the aforementioned San Juan archaeological project. This study expands on that work with additional data, and provides the first synthesis of the non-island dates from Northwestern Washington state.

In the second paragraph of the introduction the author outlines the geographic divisions for analysis, but no rationale is provided. I wonder why the radiocarbon dates from the San Juan islands are analyzed separately from the adjoining mainland? Based on historical and cultural factors the data could just as easily be grouped. I think that the geographic divisions make intuitive sense, but this could be elaborated on, especially because the relationship between the islands and the mainland seems to be importation to some of the conclusions (ie. an influx of people from the mainland to the islands).

More information has been added on the specific region research histories, how they inform the regional divisions, and why this particular study has been undertaken.

To address the comments on the relationship between the islands and the mainland, oral history accounts of the arrival to the San Juan Islands, and leaving the islands to the mainland have been added. The data appear to support these oral traditions. This has been added to the discussion.

The author added a general overview of the region with an emphasis on hereditary social inequality. It’s not clear how this all ties in with the research questions. I would have expected context relating to populations/demography, archaeological perspectives on the growth and distribution of various localized or regional populations. Or perhaps on population movements, since this is proposed in the study. Some parts of this overview would also need to be expanded on to provide adequate background information for non-regional archaeologists.

The research questions also pre-suppose that the reader has existing familiarity with the region (ie., Clark’s 2013 idea that the Fraser Delta was a regional core) and previous San Juan Island research in particular.

Providing a full literature review of the SJAP would be beyond the scope of a typical paper for this journal. However the earlier paragraphs explicitly discussing research history should address this. Additional context has been added for Clark for those not familiar with his sequence and culture-history based arguments, but described without the specific sequence building and references have been added to critiques levelled.

the first couple lines of the second paragraph of the research questions section seem unrelated to the rest of the paper.

Added connecting sentence about concerns raised regarding erosional effects on the study. Clarified that the erosional effects would not change overall trends in the study.

Methodology: It’s not clear how complete this compilation of radiocarbon dates is? This would be useful to know. Also when was the data compiled (ie. how up-to-date is it)?

Added additional clarification of the date range 1969-2022, and that the dataset includes all MAs, dissertations, gray literature, and an unpublished WWU database.

Figure 3. Many of the sites look like they are in the middle of the ocean. Must be a mapping error.

Figures have been adjusted to have a higher resolution open access shoreline from the state of Washington that includes minor islands.

Map figures: It seems important to represent the riverways, since nearly all the mainland sites are oriented to them.

Figures have been updated to include riverways from open access state of Washington data.

KDE plot figures. It’s hard for the reader to evaluate the differences between Marine vs. Terrestrial data or even the regions because the timescales on the plots are different. Ideally, they could be plotted together, with variances statistically represented.

Timescales for the figures have been adjusted, new figures have been made for each region combining the sets of unmodeled date probabilities and posterior probabilities combining the plots. The raw model data and posterior probabilities do not include variances. The reviewer is likely referring to data display from composite KDE which is not employed here which would be a potential next step, although an FMM model of specific sites is the immediate next direction.

The final figures included posterior probabilities directly comparing each region, but have been adjusted with new scales.

The results sections are all very brief and could be described more clearly so that the reader can get a better sense of how the KDE plots might relate to population dynamics.

Additional text has been added for how the posterior probabilities relate to overall population trends. Including lower population densities in the interior throughout time and how island population dynamics reflect Lummi oral traditions, at the recommendation of community members.

In the discussion the author seems to suggest that the San Juan population dynamics reflect an “influx” of people around 2,600 – 2,200 cal BP and notes that it is too simplistic to focus on resource intensification as either a driving force or result of greater populations. Because there is no discussion of demography in this manuscript, it does not seem warranted to conclude that changes in the KDE were the result of an influx of people rather than in-place demographic growth (which I would assume is the viewpoint of the Indigenous communities).

Specific oral history narratives regarding Lummi population movement have been added. However, the primary thrust has been in situ population growth and dynamism with population movement due to the nature of the marriage and social networks which the background section included but additional information tied to population dynamics was added. The addition of these oral narratives for interpretive context of the results reflects the viewpoint of the Indigenous community.

The author’s critques of other models (Croes and Hackenburger 1988 and Clark 2013) are not clearly refuted with the presented data or described in a way that can be evaluated. Also, in effect, the author seems to be describing the same sort of expansion idea, only from the Skagit River/delta to the San Juan Islands.

It was is not the point of this article to refute Croes and Hackenberger's model of general in situ demographic increase but instead noting that the literature has moved beyond using that as a social prime mover based on faunal evidence. However, the critiques of that and Clark are cited with additional context. The main thrust of this is providing a regional synthesis of dates from the gray literature and working hypotheses (now situated in Lummi oral tradition) for the trends observed.

Additionally, Lummi oral traditions note expansion from north to the Islands, and subsequent movement from the islands to the mainland. The direction of expansion is from the San Juans to the Skagit River, and appears to be reflected in the data. Similarly the discussion on hereditary inequality is important to provide context for “voting with your feet” which is a prime driver for population dynamism in the area but additional language has been added to make this explicit.

Overall the discussion should be developed and expanded so that readers can understand the contributions of the study, and how it advances other recent explorations of this same topic. One study that the author should consider is the recent Prentiss et al. 2022 study on population dynamics comparing the middle Fraser and lower Fraser regions (Divergent population dynamics in the middle to late Holocene lower Fraser valley and mid-Fraser canyon, British Columbia).

The Prentiss study is now cited, but the state of research in this particular area is less developed outside of the SJAP than the ongoing efforts by Prentiss and colleagues.

Issues with punctuation and spelling persist. There is a grammatical issue with the first sentence of the abstract.

Fixed

Reviewer #2: I am fully satisfied that all of the theoretical and substantive issues I raised in my first review have been addressed. That being said, I noted the need for some further copy editing in my initial review as well, and unfortunately some important editorial problems persist in the manuscript, including in the abstract. I will list those that I think undermine the reader experience most, in order of appearance. Because the manuscript lacks page or line numbers, I will have to use the section name and paragraph number within each section to orient the author for the purpose of correction:

Noted issues have been fixed and the manuscript was ran through additional copyediting post edits made to address reviewer #1.

Abstract: The grammar is broken in the sentence "Recent developments in radiocarbon dating have enabled archaeologists to re-examination of question of population dynamism in the Salish Sea". I suggest revising "re-examiniation of question" to "reexamine the question"

Fixed

Abstract: The words "that the" should be dropped from the following passage: "The expanded sample suggests that the patterns of population growth from 3200-2800 cal BP ...". Also, consider changing "patterns" to "a pattern"

Fixed

Section: Introduction, Para. 2: "beingutilized" should be "being utilized"

Fixed

Section: Research questions, para. 1: "If the case" should be "If this is the case"

Fixed

Section: Database, para. 1: This is a run-on sentence: "An aim of this study was to critically assess uncertainties in marine dates, shell and bone dates with marine diet contributions were included." I think the logical connector "so" needs to be added after the comma. Alternatively, change the comma to a period and begin the new sentence that follows with "Consequently."

Fixed

Section: Kernel density estimation, para. 2: typo: "methofd" should be "method"

Fixed

Section: Discussion, para. 6: Revisit the grammar and punctuation of this sentence: "While an FMM study of individual sites is more in line with previous site-level radiocarbon analyses previously done in the region, can provide new insights to specific site histories and would be complementary to a settlement pattern analysis approach."

Fixed

Additionally, I disagree with the implication that an FMM would be more inline with intra-site analysis than inter-site analyses. On one hand, it is true that finite mixture modeling is very often used as a method for model-based cluster analysis (e.g., see Bouveyrone et al. 2019 cited in my first round of review). Consequently, I would agree with the author that FMMs would be well-suited to the task of disentangling palimpsests of radiocarbon data at an intra-site level. On the other hand, FMMs can also be used simply as nonparametric density estimators. In fact, as I noted in my first review, FMM includes KDE as a special, extreme case. In this case, the mixture components are not interpreted as representing latent subpopulations in a mixed population but merely as tools for inducing flexible distributional structures in the density estimators. Consequently, FMMs are just as well suited for modeling regional dynamics in settlement patterns and demographic change as they are for disentangling latent clusters in mixed populations. This is actually the application that Price has in mind in developing the R 'bayesdem' package.

The sentence wasn't meant to imply that FMM was more appropriate for disentangling mixed populations but that it's best use for this dataset would be that type of approach. I've added a clarification sentence that it is not exclusive for the smaller scale and just as suitable.

Section: Discussion, para. 7: This sentence needs some serious editorial reworking: "2. The marine and terrestrial sample lacks some finer structure seen in terrestrial only sample but broad patterns relatively consistent, ...". Perhaps it could be revised to something like "The combined marine and terrestrial sample lacks the fine distributional structures seen in the terrestrial-only sample, but broad patterns are consistent between the two, ..."

Adjusted to recommended language.

Section: Discussion, para. 7: The word "be" is missing between "not" and "problematic" in the following passage: "3. While shell date error terms may obscure fine structure, reservoir corrections themselves may not problematic."

Fixed

7. PLOS authors have the option to publish the peer review history of their article (what does this mean?). If published, this will include your full peer review and any attached files.

If you choose “no”, your identity will remain anonymous but your review may still be made public.

Do you want your identity to be public for this peer review? For information about this choice, including consent withdrawal, please see our Privacy Policy.

Reviewer #1: No

Reviewer #2: Yes: William A. Brown
