# Supplementary material for: Assessing population dynamics in the Central Salish Sea, Pacific Northwest Coast of North America
Source: PLoS One. 2023 Aug 3;18(8):e0285021. doi: 10.1371/journal.pone.0285021 (PMC10399822; doi:10.1371/journal.pone.0285021)
Supplement: Attachment — Submitted filename: Response to Reviewers.doc [file pone.0285021.s004.doc]

Response to Reviewers:

1. If the authors have adequately addressed your comments raised in a previous round of review and you feel that this manuscript is now acceptable for publication, you may indicate that here to bypass the “Comments to the Author” section, enter your conflict of interest statement in the “Confidential to Editor” section, and submit your "Accept" recommendation.

Reviewer #1: All comments have been addressed

Reviewer #2: All comments have been addressed

2. Is the manuscript technically sound, and do the data support the conclusions?

The manuscript must describe a technically sound piece of scientific research with data that supports the conclusions. Experiments must have been conducted rigorously, with appropriate controls, replication, and sample sizes. The conclusions must be drawn appropriately based on the data presented.

Reviewer #1: Partly

Reviewer #2: Yes

3. Has the statistical analysis been performed appropriately and rigorously?

Reviewer #1: Yes

Reviewer #2: Yes

4. Have the authors made all data underlying the findings in their manuscript fully available?

The PLOS Data policy requires authors to make all data underlying the findings described in their manuscript fully available without restriction, with rare exception (please refer to the Data Availability Statement in the manuscript PDF file). The data should be provided as part of the manuscript or its supporting information, or deposited to a public repository. For example, in addition to summary statistics, the data points behind means, medians and variance measures should be available. If there are restrictions on publicly sharing data—e.g. participant privacy or use of data from a third party—those must be specified.

Reviewer #1: Yes

Reviewer #2: Yes

5. Is the manuscript presented in an intelligible fashion and written in standard English?

PLOS ONE does not copyedit accepted manuscripts, so the language in submitted articles must be clear, correct, and unambiguous. Any typographical or grammatical errors should be corrected at revision, so please note any specific errors here.

Reviewer #1: (No Response)

Reviewer #2: Yes

6. Review Comments to the Author

Please use the space provided to explain your answers to the questions above. You may also include additional comments for the author, including concerns about dual publication, research ethics, or publication ethics. (Please upload your review as an attachment if it exceeds 20,000 characters)

Reviewer #1: Overall, I find that this manuscript has improved considerably from the original submission, but there are still a few changes/additions that would ideally be addressed before publication.

One issue is that there is no discussion on population growth overall, starting with radiocarbon evidence for people living in inland, then on the coast, then on the islands. I’m not clear why there is no real mention of the broader trends as indicated by the radiocarbon dates. The discussion focuses-in on only a couple peaks (mostly related to the Islands). This is fine of course, but it’s not explained why some peaks are emphasized more than others, or over the general trends.

An additional paragraph outlining the demographic work done in the 1980s and 1990s has been added and a new figure with the broader trends for the entire Salish Sea. Specific callouts to the emphasized peaks diverging from the "regional demographic model" have been added. An additional sentence in the discussion about multi-scalar analyses being important that combining current data sets shows a general curve consistent with the older models, but obscuring regional structure, has also been added.

At one point the author notes that there is in-situ population growth in NW coastal Washington between 3,200 – 2,800 cal BP. People were living in this area long before this time though…was this earlier time characterized by something other than in-situ growth? I think that this is only confusing because there is not a more general review discussion on longer-term population trends.

There is no evidence of earlier population growth not being in situ in that region, the sentence has been updated to state "continuing in situ population growth" for clarity

In the San Juan results section the first peak is described as being between 2,400 – 2,200 cal BP.

In the discussion it’s reported as starting at 2,600 cal BP. Which is more accurate?

The discussion and abstract are correct in that it is 2,600 cal BP to 2,200 cal BP. 2400-2200 is the NW WA sample the text has been adjusted accordingly.

So, even though people have been living on the San Juan islands since before 3,000 years ago, the argument is that the First Ancestor of the Lummi people may have arrived around 2,600/2,400 cal BP because there is an increase in population at this time?

That was not the intent of the statements and so text has been clarified that oral tradition supports the initial in situ population growth and the later movement to the mainland. If there was other large scale population movement from the mainland to the islands, beyond the noted village fission-fusion dynamics in the background and discussion, it is not supported at this time based on current evidence. The text has been adjusted to avoid readers making that misconception. The Island to mainland movement is supported by oral tradition and so the text has been adjusted further to clarify that.

I also wonder about the word choice of “influx” to describe this significant event in tribal history. Influx makes it sound like the First Ancestor is coming from somewhere nearby (like the mainland, as was suggested in the second version). Typically though, these oral histories are explicit about the ancestors coming from the sky, and that they are the original settlers in a new area.

In this case influx is appropriate for the Lummi to the mainland but the language in the abstract and discussion has been altered to "population growth" in the San Juans to be more consistent with oral traditions that the same ancestor in the San Juan Islands was an ancestor to populations in SE Vancouver Island. Additionally more information from place names has been added to note that these apical ancestors “appeared or came down” in a manner not unlike what Reviewer #1 suggests.

The figures are better than before, but could still be improved with place names (ie., coastal and riverine water bodies) to help orient the reader.

Major riverine bodies of relevance (Nooksack River, Skagit River, Fraser River have been added. Strait of Juan de Fuca and Strait of Georgia also added.

Figure 1. “Stolo” is the only name provided for a cultural group on the Canadian side of the map, and it’s use is inaccurate. I suggest sticking with linguistic groupings at this geographic scale.

Additional referred to groups in text have been added to address this concern as well as the Musqueam to match with the Suttles reference.

Reviewer #2: (No Response)

7. PLOS authors have the option to publish the peer review history of their article (what does this mean?). If published, this will include your full peer review and any attached files.

If you choose “no”, your identity will remain anonymous but your review may still be made public.

Do you want your identity to be public for this peer review? For information about this choice, including consent withdrawal, please see our Privacy Policy.

Reviewer #1: No

Reviewer #2: Yes: William Brown

[NOTE: If reviewer comments were submitted as an attachment file, they will be attached to this email and accessible via the submission site. Please log into your account, locate the manuscript record, and check for the action link "View Attachments". If this link does not appear, there are no attachment files.]

While revising your submission, please upload your figure files to the Preflight Analysis and Conversion Engine (PACE) digital diagnostic tool, https://pacev2.apexcovantage.com/. PACE helps ensure that figures meet PLOS requirements. To use PACE, you must first register as a user. Registration is free. Then, login and navigate to the UPLOAD tab, where you will find detailed instructions on how to use the tool. If you encounter any issues or have any questions when using PACE, please email PLOS at figures@plos.org. Please note that Supporting Information files do not need this step.

In compliance with data protection regulations, you may request that we remove your personal registration details at any time. (Remove my information/details). Please contact the publication office if you have any questions.
